# A Universal and Versatile Zwitterionic Coating for Blood‐Contacting Catheters with Long Lengths and Complex Geometries

**DOI:** 10.1002/advs.202502411

**Published:** 2025-03-24

**Authors:** Tong Zhang, Tian Liang, Qichao Pan, Shouyan Zhang, Shuhua Zhang, Zhi Geng, Bo Zhu

**Affiliations:** ^1^ School of Materials Science and Engineering Shanghai University 99 Shangda Road Baoshan Shanghai 200444 China; ^2^ Shanghai Engineering Research Center of Organ Repair Shanghai University Shanghai 200444 China; ^3^ Joint International Research Laboratory of Biomaterials and Biotechnology in Organ Repair Ministry of Education Shanghai University Shanghai 200444 China

**Keywords:** anticoagulant, antifouling, catheter, phosphorylcholine, spontaneous coating

## Abstract

Blood‐contacting catheters are highly susceptible to thrombus formation, making heparin coating essential for reducing clinical complications. However, the limitations of heparin coatings have spurred significant efforts to develop alternative strategies. This study demonstrates a cost‐efficient, mechanically viable, and universal zwitterion coating approach for long and complex catheters with near‐zero fouling, super anticoagulation, and selective biocapturing. Leveraging the synergistic action of side groups, a wet‐adhesive initiator‐bearing polymer rapidly assembles on catheter surfaces in aqueous environments, facilitating the grafting of superhydrophilic and zwitterionic polymers onto catheter inner walls. This strategy demonstrates broad adaptability, successfully applying to ten substrates and showing exceptional versatility in modifying catheters and joints of various shapes and sizes. These coatings exhibit near‐zero protein fouling across a broad pH range, and superior resistance to blood cells and bacteria. Furthermore, they maintain excellent stability under simulated bloodstream without compromising anticoagulant performance. Beyond antifouling properties, this method enables the construction of highly selective bio‐interaction networks on catheter inner walls, allowing precise capture of circulating tumor cells from blood. This zwitterion coating technique, with its rapid modification, robust anticoagulant properties, and customizable bio‐functionality, provides an attractive solution for, beyond catheters, a wide range of medical devices that must perform in challenging biological environments.

## Graphical abstract

A universal and versatile zwitterion coating strategy is developed for blood‐contacting catheters with long lengths and complex geometries. The coating exhibits near‐zero fouling, superior anticoagulation, and selective biocapturing properties while maintaining excellent stability under dynamic blood flow. This robust and scalable approach offers a versatile solution for advanced medical catheter applications.

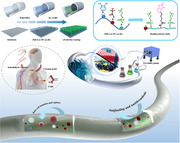

## 1 Introduction

Catheters of various shapes and sizes have widely served as blood‐contacting components in medical devices such as artificial lungs,^[^ ^1^ ^]^ ventricular assist devices,^[^ ^2^ ^]^ hemodialysis catheters,^[^ ^3^ ^]^ extracorporeal membrane oxygenation (ECMO),^[^ ^4^ ^]^ and vascular grafts of some clinical medical instruments, saving countless lives. However, due to the complexity of human blood, when blood is introduced into a tube, the proteins and platelets nonspecifically bind to the inner wall, leading to thrombus formation and inflammation. The detachment and migration of blood clots would further cause complications, such as pulmonary embolism, deep vein thrombosis, heart attack, and vascular access failure. In particular, the risk of complications would become much higher in small‐sized tubular devices.^[^ ^5^, ^6^ ^]^ For example, the hemodialyzer usually contains many microtubules with a much larger surface area, leading to extensive thrombus formation.^[^ ^7^ ^]^ Long‐term use of central venous catheters was reported to have a higher incidence of vascular access failure than 66% when used to treat hemodialysis patients.^[^ ^8^ ^]^ In addition, the formation of thrombi inside microtubules has been demonstrated to decrease by 33% the survival rates of the patients treated by ECMO.^[^ ^9^ ^]^

Constructing anticoagulant coatings for these devices is considered one of the most straightforward approaches to improving blood compatibility and reducing thrombus formation and inflammation. Heparin, a natural polysaccharide, has been commonly used as an efficient anticoagulant material for tubular devices because of its unique ability to activate antithrombin and inhibit coagulation factors.^[^ ^10^, ^11^, ^12^ ^]^ Since Gott discovered the anticoagulant feature of heparin in 1963,^[^ ^13^ ^]^ many heparin‐based surface modification methods have been developed to construct anticoagulant surfaces for blood catheters, artificial hearts and lungs, and vascular stents.^[^ ^14^, ^15^ ^]^ However, some terrible limitations are associated with the heparin coating techniques.^[^ ^16^ ^]^ The heparin coating can prevent thrombus formation on the surface by activating antithrombin, whereas, unfortunately, it also increases the risk of malignant bleeding when heparin molecules are released from the heparin coating.^[^ ^17^, ^18^ ^]^ In addition, prolonged exposure to heparin molecules was found to be responsible for the development of heparin‐induced thrombocytopenia, which is caused by an immune response triggered by heparin binding to the platelet at the platelet factor 4. It typically causes a significant drop in platelet count, with mortality and disability rates as high as 30%.^[^ ^19^ ^]^ Particularly, the negatively charged heparin coating would electrostatically adsorb some positively charged plasma proteins, reducing and even taking all the catalytic sites of the coating.^[^ ^20^, ^21^ ^]^ These critical limitations of heparin coating have driven urgent efforts to develop alternative strategies.

Hydrophilic polymers strongly interact with water molecules through hydrogen bonding or electrostatic interaction and thus form a tightly bound hydration layer to prevent the nonspecific adsorption of proteins and biological components. Its application as a catheter coating can effectively prevent the initiation of thrombus formation at its earliest stages. The zwitterionic polymers, containing phosphocholine, carboxybetaine, or sulfobetaine groups, strongly interact with water molecules through electrostatic interaction, resulting in more robust hydration layers and, thus, stronger resistance to protein adsorption.^[^ ^22^, ^23^, ^24^ ^]^ Among them, the phosphorylcholine groups are particularly interesting, as they are one of the main components of cell membrane phospholipids, featuring excellent biocompatibility.^[^ ^25^ ^]^ Additionally, its inner salt structure and the balanced charge density of the cation and anion ensure its electrical neutrality, thus significantly eliminating its electrostatic interaction with charged biomolecules or cells.^[^ ^26^ ^]^ Furthermore, the weak basicity of the trimethylamine group and the stable phosphate structure endow the phosphorylcholine group with excellent resistance to protonation and deprotonation, ensuring its electrical neutrality over a wide pH range (≈4–10).^[^ ^27^ ^]^ All these compelling attributes make the phosphorylcholine‐functionalized polymer an ideal coating material for medical catheters, providing outstanding antifouling and anticoagulant properties while minimizing the risk of adverse side effects. Remarkably, recent attention has increasingly centered on preparing phosphorylcholine‐functionalized polymer coatings using the graft‐from method rather than the graft‐to technique.^[^ ^28^ ^]^ This approach results in densely packed zwitterionic polymer chains and a more resilient hydration layer, which are critical for achieving near‐zero protein adsorption and significantly enhanced anticoagulant properties.^[^ ^29^ ^]^ However, the current techniques for grafting zwitterionic polymers from surfaces face significant challenges in terms of compatibility with medical catheters, especially those with long, narrow, and complex shapes, primarily due to their difficulty in activating the inner walls of catheters for grafting zwitterionic polymers from the surface. Moreover, these techniques are typically cost‐ineffective, poor substrate‐adaptable, and time‐consuming, as they require complex surface activation. These limitations currently hinder their practical application in the fabrication of anticoagulant catheters.

The supramolecular anchoring methods utilizing *π*–*π*, hydrogen‐bonding, hydrophobic‐hydrophobic, and electrostatic interaction can remove the limitations of the current zwitterion grafting approaches with their activation‐free anchoring capability.^[^ ^30^, ^31^ ^]^ Recent progress implies that some supramolecular anchoring‐assisted coating approach might demonstrate activation‐free anchoring capability and the antifouling property. For example, dopa‐conjugated Atom Transfer Radical Polymerization (ATRP) initiators can directly anchor onto metal,^[^ ^32^, ^33^, ^34^, ^35^ ^]^ metal oxide,^[^ ^36^, ^37^, ^38^ ^]^ glass,^[^ ^39^ ^]^ silica,^[^ ^40^ ^]^ poly(vinylidene fluoride),^[^ ^41^ ^]^ poly(ethylene glycol terephthalate) (PET),^[^ ^42^ ^]^ polypropylene,^[^ ^22^, ^43^ ^]^ poly(dimethylsiloxane) (PDMS),^[^ ^32^, ^43^ ^]^ Polytetrafluoroethylene.^[^ ^44^ ^]^ They subsequently initiate the growth of a high‐density zwitterionic polymer. This activation‐free zwitterion grafting provides a more robust zwitterion coating, as the dopa‐substrate interaction, as revealed by single‐molecule spectroscopy,^[^ ^45^ ^]^ involves a variety of dynamic and diverse interaction modes. Additional aspects that facilitate the activation‐free zwitterion grafting approach include the recent advances in addressing the challenges of ATRP in aqueous solvents, such as the dissociation of deactivator complexes, decomplexation of the catalyst, and disproportionation of catalyst complexes.^[^ ^46^, ^47^, ^48^ ^]^ Despite the attractive progress in activation‐free anchoring and controllable polymer growth, the present dopa‐assisted zwitterion grafting method suffers from some intrinsic issues, including a slow anchoring process and weak substrate interactions. These issues significantly compromise the cost‐efficiency, applicability, and reliability of the technique. Among these limitations, the slow anchoring process of the current dopa‐assisted coating approach is one of the most detrimental factors preventing its application in catheters, as a time‐consuming modification procedure exceeding 12 h is impractical for commercial medical catheter production.^[^ ^49^, ^50^, ^51^ ^]^ In addition, the robustness of the zwitterion coating is another critical concern for this dopa‐assisted approach, as the intense hydration likely extends the zwitterionic polymer chains and thus loads a mechanical press on this supramolecular anchoring.^[^ ^52^ ^]^ Therefore, there is a pressing need to develop a more cost‐efficient and viable strategy for depositing zwitterion coatings that offer broad substrate adaptability and excellent catheter compatibility. Moreover, current approaches to catheter modification, have not demonstrated the ability to endow hydrophilic catheter coatings with specific bio‐functions (e.g., biorecognition units, anticoagulant groups, and antibacterial factors) in addition to antifouling properties, primarily due to the complexities associated with present zwitterion graft‐to methods. Addressing this limitation is also very critical, as these bio‐functional coatings are essential for enhancing blood compatibility or removing harmful proteins and cells in blood‐contacting catheters.^[^ ^53^, ^54^, ^55^ ^]^

**Figure 1:**
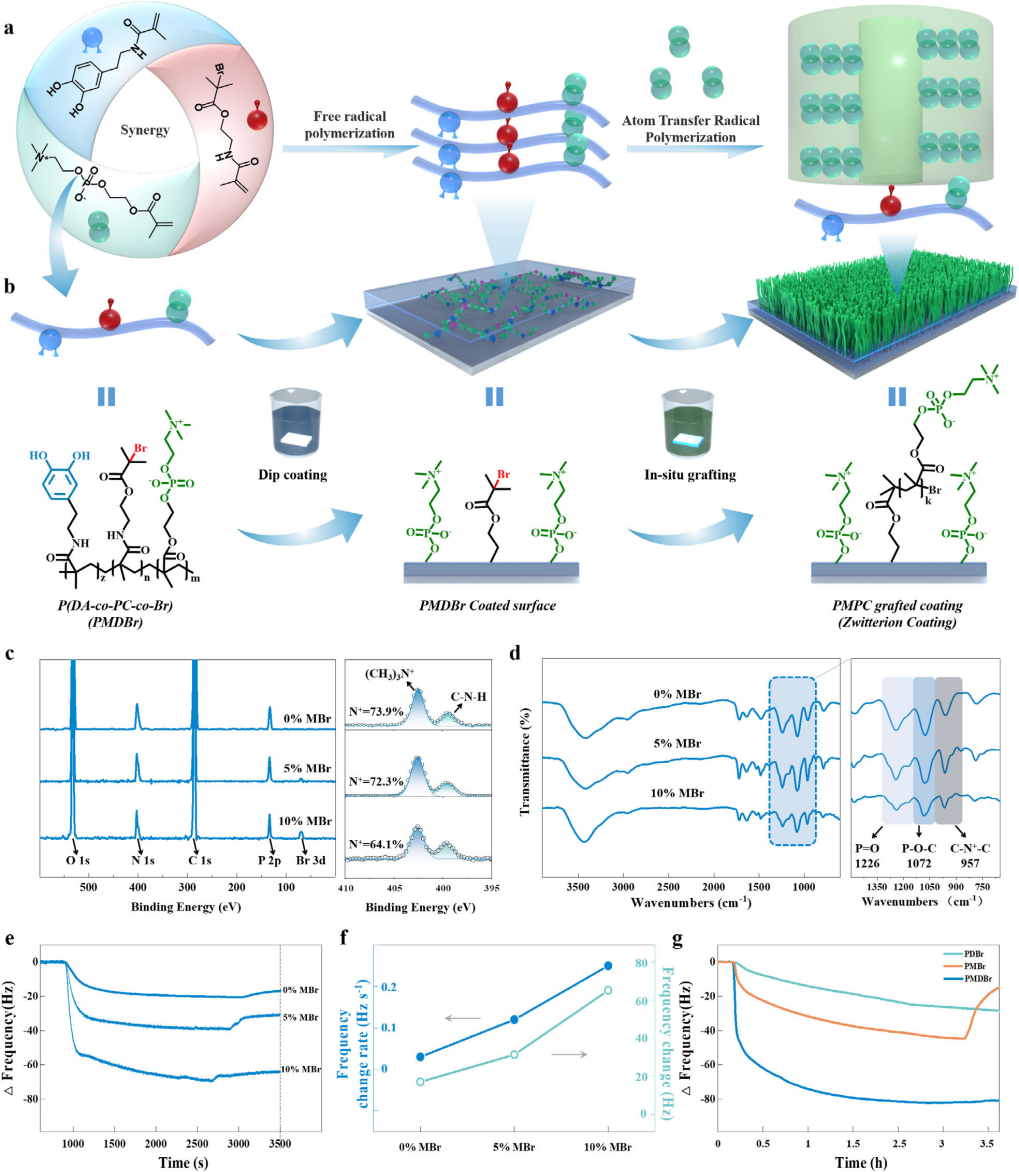
A quick, spontaneous, and substrate‐independent approach to graft zwitterionic polymer. a,b) Schematic representation of the synthesis of an adhesive polymer with phosphorylcholine, dopa, and bromoisobutyrate side groups (PMDBr), its spontaneous binding to substrates in the aqueous solution during dip coating for immobilizing 2‐bromoisobutyrate groups on substrates, and grafting phosphorylcholine functionalized polymer chains from substrates via SI‐ATRP. c) XPS survey spectra (left) and High‐resolution XPS spectra of N 1s (right) for the PMDBr synthesized at varied MBr compositions (0%–10%). d) FT‐IR survey spectra for the PMDBr synthesized at varied MBr compositions (0%–10%). e) In situ monitoring of the frequency change of gold QCM crystals resulted from the binding of the PMDBr synthesized at varied MBr compositions (0%–10%) in their solutions in an aqueous solvent (DMSO/H_2_O, v/v, 1/1) at a concentration of 1.5 mg mL^−1^. f) The plots of the QCM frequency change rate and the frequency change (Δf) for the PMDBr synthesized at varied MBr compositions (0%–10%). g) In situ monitoring of the frequency change of gold QCM crystals resulted from the binding of the PDBr (25% DMA and 75% MBr), PMBr (90% MPC and 10% MBr), and PMDBr (65% MPC,25% DMA and 10% MBr) with crystals.

In this study, we developed a universal and versatile zwitterion coating method for modifying the blood‐contacting catheters with complex geometries and long lengths, featuring cost‐efficiency, mechanical viability, activation‐free process, and broad substrate‐adaptability (**Figure** **1**a,b). We synthesized, for the first time, an aqueous‐soluble and wet‐adhesive ATRP‐initiator‐bearing polymer, leveraging the synergistic action of side groups to enable fast and robust assembly on catheter surfaces in an aqueous environment and uniform grafting of zwitterionic polymers from the inner walls of long and complex catheters. This approach effectively harnessed the synergistic action of side groups to remove the key limits, i.e., slow immobilization and weak substrate interaction, of the present dopa‐assisted method. The phosphorylcholine side groups solubilize this polymer in aqueous solutions and dramatically enhance polymer‐substrate interaction, enabling an intense assembly process in an aqueous environment. Additionally, this aqueous assembly process further takes advantage of the hydrophobic force of the bromoisobutyrate side groups, promoting the surface assembly of the polymer. In this way, the side group synergistic strategy can accelerate the aqueous assembly rate by nearly 44 times, enabling modification of various substrates within 3 min, far faster than the ≥ 12 h required by conventional methods. Thus‐prepared zwitterion‐coated surfaces exhibit near‐zero protein fouling within a broad pH range and superior resistance to diverse blood cells and bacterial. This approach is further verified as a robust and scalable solution for applying exceptionally effective anticoagulant coatings to a broad range of catheters with long lengths, narrow sizes, and complex shapes. The resulting anticoagulant coating demonstrated its excellent stability and durability without compromising its anticoagulant performance under a 7‐day simulated bloodstream. Furthermore, it facilitates constructing a highly selective bio‐interaction network on catheter inner walls, enabling the precise and efficient capture of circulating tumor cells from the bloodstream. We believe this side group synergistic approach presents an efficient and innovative solution for engineering the inner lumens of medical catheters, offering rapid modification, robust anticoagulation, and customizable bio‐functionality to comprehensively meet diverse clinical demands for advanced medical catheters.

## 2 Results and Discussion

### 2.1 Wet Adhesive Initiator‐Bearing Polymer with Synergistic Side Group Action

To effectively address the challenges associated with current supramolecular anchoring methods, we aimed to synthesize a new linear polymer bearing dopa, phosphorylcholine, and bromoisobutyl side groups through radical polymerization (Figure 1a). The high composition of phosphorylcholine side groups was incorporated not only to replicate the antifouling properties of cell membranes but also to solubilize the polymer into an aqueous solution and substantially enhance the polymer‐substrate interaction, as revealed later. However, these dopa moieties readily react with the propagating radicals through the hydrogen abstraction of the phenolic hydrogen, and these generated radicals subsequently combinate with other propagating radicals to form branched and cross‐linked structure, making it challenging to prepare a soluble dopa‐contained copolymer through classical radical polymerization.^[^ ^56^, ^57^, ^58^ ^]^ As this chain‐transfer event depends on the nature of the propagating radical, we chose methyl methacrylate or methyl methacrylamide as the backbone build block to prepare this copolymer.^[^ ^56^, ^59^ ^]^ Additionally, we employed a DMF‐rich solvent (DMF/H_2_O (v/v, 4/1)), strongly hydrogen‐bonding dopa groups, to further depress the reactivity of dopa groups.^[^ ^60^ ^]^ In this way, we successfully synthesized a series of copolymers with compositions quantitatively aligned with the monomer ratios in the reaction medium, as estimated by the liquid ^1^H NMR (Figure S2 and Table S1, Supporting Information). The X‐ray photoelectron spectroscopy (XPS) and Fourier‐transform infrared spectroscopy (FTIR) results further verified this composition's dependence (Figure 1c,d). Typically, three copolymers (PMDBr) were synthesized with a fixed Dihydroxyphenyl)ethyl methacrylamide (DMA) composition (25%) and varied 2‐methacryloyloxyethyl phosphorylcholine (MPC) (75%, 70%, and 65%) and 2‐(2‐bromoisobutyryl) ethyl methacrylamide (MBr) (0%, 5%, and 10%) compositions for subsequent experiments. All these copolymers exhibit good solubility in an aqueous solvent (DMSO/H_2_O, v/v, 1/1), indicating the absence of the cross‐linked polymers. In addition, the copolymer of DMA and MBr (PDBr) and that of MPC and MBr (PMBr) were similarly synthesized as controls. PDBr and PMBr were soluble in DMSO and water, respectively. From now on, these solution conditions were applied in experiments to evaluate their interaction with various substrates.

To investigate the polymer‐substrate interaction, we used quartz crystal microbalance (QCM) to monitor the assembly of PMDBr on a gold substrate in an aqueous environment, using the frequency change rate to define the assembly rate. The results indicate that the assembly rate of PMDBr increased from 0.03 to 0.25 Hz s^−1^ as the MBr monomer composition rose from 0% to 10% (Figure 1e), causing the polymer's assembly process to reach saturation within 3 min. Additionally, the saturated frequency change, corresponding to the mass of the polymers bound to the surface, increased in parallel with the MBr composition (Figure 1f), indicating that a thicker PMDBr layer was formed at higher MBr compositions. The assembly layer thicknesses were estimated to be 1.9, 3.6, and 7.3 nm for the PMDBr copolymers with compositions of 0%, 5%, and 10%, respectively. It is well aligned with the XPS results for the coatings on the silicon wafer, where the Si signals (152.08 and 101.08 eV) gradually diminished with the MBr composition increasing (Figure S3, Supporting Information).

To clarify the roles of the three side groups in the polymer assembly process, we compared the surface assembly dynamics between the PMDBr (10% MBr) and the PMBr (10% MBr) (without dopa side groups) (Figure 1g; and Table S2, Supporting Information). The results clearly indicated that the dopa side groups drive the copolymer to bind to the gold surface, ensuring the polymer firmly adheres to the surface and remains intact after washing. Additionally, comparing the surface assembly dynamics between the PMDBr (10% MBr) and the PDBr (75% MBr) (without MPC groups) revealed that incorporating phosphorylcholine side groups significantly accelerates and strengthens the surface assembly of polymers (Figure 1g; and Table S2, Supporting Information). PMDBr achieves an assembly rate of 0.25 Hz s^−1^ and a saturated frequency change of 65.17 Hz, 44 times and 8 times higher than PDBr. Its underlying mechanism is not entirely unclear but may be related to the good solubility of dopa groups and the protonation of amine groups in the aqueous environment.^[^ ^61^ ^]^ Considering the dependence of PMDBr assembly on the MBr composition (Figure 1f), it is evident that incorporating bromoisobutyrate side groups further amplifies the polymer‐substrate interaction by introducing hydrophobic forces, although these side groups alone do not induce any polymer‐substrate interaction without the presence of dopa side groups (Figure 1g; and Table S2, Supporting Information). The synergistic action of the three side groups can accelerate the surface assembly of polymer to reach saturation in only 3 min, far faster than the ≥12 h required by the current methods. Therefore, the synergistic strategy of the side groups effectively addresses the key limitations, i.e., slow immobilization and weak substrate interaction, of the current dopamine‐assisted method and holds great potential as a rapid and cost‐efficient approach for modifying the inner walls of long and complex catheters without surface activation.

### 2.2 Fast and Universal Zwitterion Coating Approach

**Figure 2:**
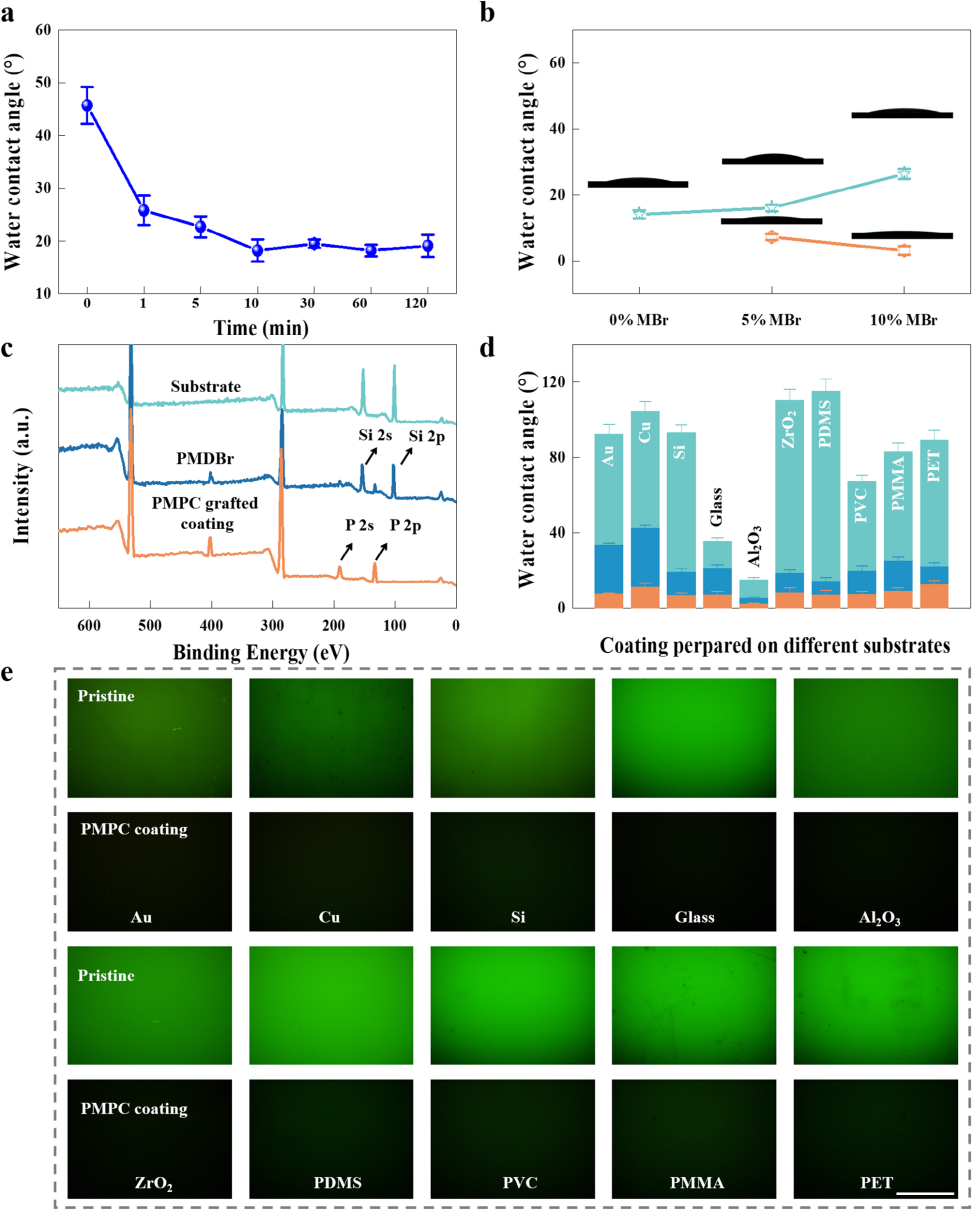
Activation‐Independence, and Broad‐Substrate‐Adaptability of Zwitterion Coating. a) The surface contact angle change of the silicon substrate after immersing it in the aqueous PMDBr solution for different durations. b) The water contact angles of the substrates coated with the PMDBr synthesized at 0%, 5% and 10% MBr compositions (green) and those further grafted by PMPC chains (orange). The water contact angle was calculated by averaging results measured by three parallel samples. c) XPS survey spectra of the original Si substrates (green), those coated with the PMDBr synthesized at 10% MBr composition (blue), and those further grafted with poly (2‐methacryloyloxyethyl phosphorylcholine) (PMPC) (orange). d) The water contact angles of the Au, Cu, Si, glass, Au, Cu, Si, Al_2_O_3_, ZrO_2_, Glass, PDMS, PVC, PMMA, and PET substrates (green), and those coated with the PMDBr synthesized at 10% MBr compositions (blue) and those further grafted by the PMPC chains (orange). e) Fluorescence microscopic images after exposure to the FTC‐BSA solutions for the various substrates before and after coating by PMPC. The scale bar is 150 µm.

To further verify it, we monitored the surface contact angle change of the silicon substrate after immersing it in the aqueous PMDBr solution for different durations (0, 1, 5, 10, 30, 60, and 120 min) without string (**Figure** **2**a). The contact angle decreased with time but reached saturation (≈19°) at 10 min, indicating that spontaneous assembly occurs very fast. We also observed that the polymer requires a slightly longer time to bind the surface in the absence of solution flow, compared to the QCM experiment.

The saturated contact angles of the PMDBr‐coated substrates increase with the MBr composition due to the hydrophobic feature of the bromoisobutyrate (Figure 2b). Notably, all the PMDBr‐coated substrates presented a significant decrease in the contact angle after exposure to the reaction medium for grafting hydrophilic PMPC polymers. The XPS results for the coated substrate with 10% MBr further confirm the modification process (Figure 2c). The contact angles of the PMPC‐grafted substrates decrease with the MBr composition increasing. Remarkably, the coated substrate with 10% MBr reaches a superhydrophilic state (≈4°), smaller than that (≈7°) of the coated substrate with 5% MBr, indicating the former may have improved antifouling potential. This phenomenon should be attributed to its more tightly packed polymer chains, as indicated by its much smoother surface (Rq = 5.33 nm) than the coated substrate with 5% MBr (Rq = 14.0 nm) (Figure S5, Supporting Information). Therefore, the 10% MBr composition is likely more suitable for achieving a superior antifouling and anticoagulant property, as both superhydrophilicity and dense packing polymer chains are critical for preventing biofouler from approaching. Considering the much more promoted assembly for the PMDBr with 10% MBr revealed above, we prefer to use its rapid spontaneous assembly to develop a highly efficient and activation‐free approach to grafting PMPC polymer chains from diverse substrates.

To evaluate its substrate adaptability, we then used it to modify ten available substrates (including the silicon wafer) simultaneously. All the tested substrates (Au, Cu, Si, Glass, Al_2_O_3_, ZrO_2_, PDMS, polyvinyl chloride (PVC), polymethacrylate (PMMA), and polyethylene terephthalate (PET)) presented a much‐decreased water contact angle after being immersed in the PMDBr (65% MPC, 25% DMA, and 10% MBr) solution for 10 min. Following grafting with PMPC chains, all the substrates achieved superhydrophilicity with a water contact angle of ≈10° (Figure 2d). XPS measurements confirmed the successful grafting of PMPC polymer chains on all ten substrates, with consistent atomic ratios of C, N, O, and P (Figures S6 and S7, and Table S4, Supporting Information), demonstrating its broad substrate adaptability and uniformity. We also exposed the above‐coated ten substrates to the fluorescein‐isothiocyanate‐labeled bovine serum albumin (FITC‐BSA) buffer to visualize the plausible biofouling spatially. Again, no fluorescence signal could be observed on any region of coated substrates (Figure 2e), indicating the superior antifouling capability and the spatial uniformity of this coating and further confirming its reliability and broad substrate adaptability. Therefore, the spontaneous assembly of the wet adhesive polymer likely offers a zwitterion coating approach that provides a rapid, efficient, and adaptable solution for modifying medical catheters across diverse substrates.

### 2.3 Near‐Zero Biofouling and Super Anticoagulation of Zwitterion Coating

**Figure 3:**
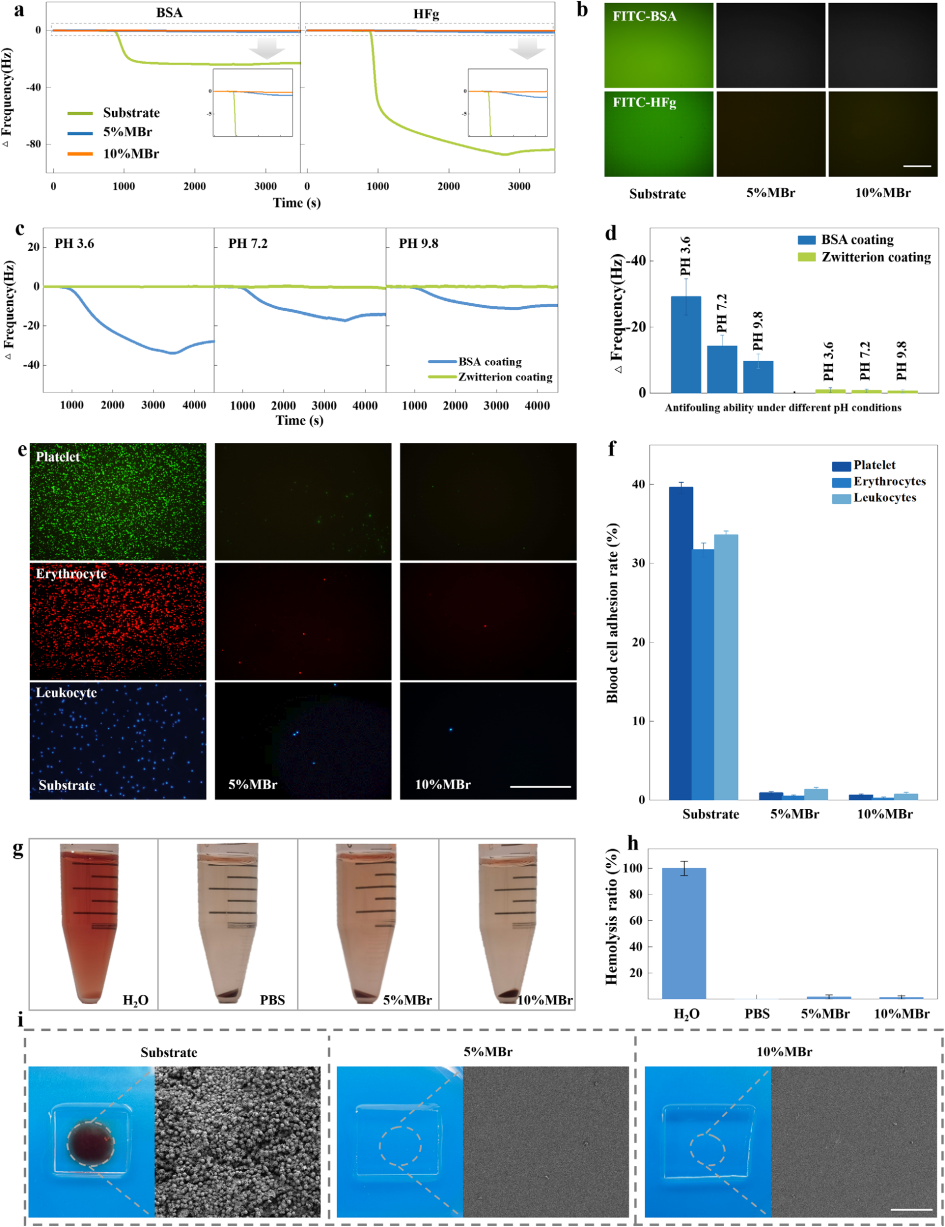
Low cell toxicity and robust antifouling and anticoagulation of zwitterionic polymer coating. a) In situ monitoring of the frequency change (Δf) resulted from the nonspecific BSA (left) and HFg (right) adsorptions of zwitterion coatings prepared from PMDBr synthesized at 5% and 10% MBr compositions. The uncoated gold QCM crystal was included as a control. b) Fluorescence microscopic images after exposure to the FTC‐BSA and FTC‐HFg solutions for the pristine glass coverslip and those with zwitterion coating prepared by PMDBr synthesized at 5% and 10% MBr compositions. The scale bar is 150 µm. c) In situ monitoring of the Δf induced by nonspecific interaction of the zwitterion (green) and the BSA (blue) coatings in the HFg solutions at various pH values, and d) the summary of the Δf of the two coating after thoroughly exposed to the HFg solutions at various pH values. e) Fluorescence microscopic images, after seeded platelets (green), erythrocytes (red), and leukocytes (blue) for 3 h, for the pristine glass coverslip and those with zwitterion coating prepared by PMDBr synthesized at 5% and 10% MBr compositions. The scale bar is 300 µm. f) Cell adhesion rates of platelet, erythrocytes, and leukocytes on the pristine glass coverslip and zwitterion coatings. The bars represent the mean ± SD (*n* = 3). g) Optical image of erythrocytes dispersed in the DI water, the PBS buffer, and the leachates of the zwitterion coatings. h) Hemolysis ratio measured in the DI water, the PBS buffer, and the leachates of the zwitterion coatings. i) Optical image and SEM image of blood clot formation on pristine glass coverslips and those with zwitterion coating prepared from PMDBr synthesized at 5% and 10% MBr compositions. The scale bar is 50 µm.

As is well known, once in contact with blood, the inner walls of catheters immediately adsorb plasma proteins and blood cells through nonspecific interaction, triggering the first step toward blood coagulation. The coagulation issue for the blood‐contacting materials may be addressed thoroughly if this zwitterion coating can prevent the nonspecific binding of proteins and cells. This zwitterion coating could significantly depress the nonspecific binding of bovine serum albumin (BSA) and human fibrinogen (HFg) of the substrate, as the zwitterion coating offers a dense hydration layer that inhibits the adhesion of proteins on the surface (**Figure** **3**a). Their resistance to nonspecific binding measured by QCM varies slightly with the grafting density (the MBr composition) of PMPC chains. An evaluation by exposing the coating to FITC‐BSA and the fluorescein isothiocyanate‐labeled human fibrinogen (FITC‐HFg) buffers further confirmed the superior performance of coatings in resisting the nonspecific protein binding (Figure 3b). In addition, no fluorescein signal could be detected in any place on the coated surface, indicating the uniformity of this coating approach. The coating with 10% MBr features a more robust antifouling property, as evidenced by its almost zero BSA and HFg binding measured by the QCM experiment, further strengthening our preference for developing an anticoagulant coating based on the PMDBr with 10% MBr.

It is also interesting to investigate the plausible pH dependence of the antifouling property of this coating. As investigated by QCM, the PMPC grafted zwitterion coating retains its HFg resistance in both acidic and basic conditions, featuring ultralow protein fouling within an extensive pH range (Figure 3c,d). In contrast, the resistance of the BSA coating (a gold standard for minimizing nonspecific protein interaction on surfaces) control to the nonspecific binding of HFg entirely depended on the pH value, with significantly compromised antifouling property in the acid environment, likely attributed to its enhanced electrostatic attraction under low pH conditions.^[^ ^62^ ^]^ This pH‐independent behavior of this PMPC‐based zwitterion coating is attributed to the weak basicity of the trimethylamine group and the stable phosphate structure, which endow the phosphorylcholine group with excellent resistance to protonation and deprotonation and ensure its electrical neutrality over a wide pH range. It has been reported that phosphorylcholine coatings steadily maintain a nearly zero zeta potential, even after prolonged exposure to acidic or alkaline environments.^[^ ^63^, ^64^ ^]^

We further exposed this coating to platelets, erythrocyte cells, and leukocyte cells to evaluate their resistance to the nonspecific interaction of blood cells (Figure 3e). All the coatings present a robust resistance to all the tested blood cells compared to the uncoated substrate, as almost no blood cell could attach to these coatings, whereas thousands of cells could be observed on the pristine substrate. We further measured the substrate area covered by cells and calculated the cell adhesion rate by dividing it using the whole substrate area (Figure 3f). A lower cell adhesion rate could be observed for the coating with 10% MBr, indicating its superior resistance to nonspecific interaction, consistent with the protein resistance results.

To evaluate the cell compatibility of this coating, we cultured the mouse embryonic fibroblast cell line (NIH3T3 cells) in a medium extracted with a coating sample (prepared using the PMDBr with 10% MBr) and monitored their cell proliferation behavior. The cell densities of NIH3T3 cells cultured in the extracted medium were compared to those cultured in a standard medium (Figure S10a, Supporting Information). The results demonstrated that the cell densities of the two samples are comparable throughout the cell culture experiment without significant differences, indicating this zwitterion coating is compatible with the NIH3T3 cells (Figure S10b, Supporting Information). The live‐dead staining experiment indicated that NIH3T3 cells maintained over 95% cell viability after 72 h of cultivation in the zwitterion coating extracts (Figure S10c, Supporting Information), confirming their biocompatibility.

We then investigated the blood compatibility of these coatings by evaluating their hemolysis ratios (HR). To measure the HR values of the zwitterion coatings, we exposed the erythrocyte cells to the medium extracted with the zwitterion coating and measured the medium supernatant absorption at 540 nm associated with the hemoglobin, which was released by the broken erythrocyte cells^[^ ^65^ ^]^ (Figure 3g). The HR values of two zwitterion coatings are below 2%, which is the safe value for biomaterials as declared by the ISO 10993–4 standard. In detail, the HR value (1.23%) for the PMPC coating with 10% MBr is smaller than that (1.61%) for the PMPC coating with 5% MBr (Figure 3h). In addition, the anticoagulant properties of the two coatings were investigated by evaluating the coagulation formation on their surfaces after 60 min of blood exposure. No clotting could be observed on the two PMPC‐coated samples, in sharp contrast to the pristine glass coverslip (Figure 3i). Furthermore, under scanning electron microscopy (SEM) observation, no clotting evidence could be found even at the microscale for the two zwitterion coatings, verifying their superior anticoagulation property again.

Therefore, all the above results support the excellent blood compatibility of the two zwitterion coatings, implying both are promising for engineering blood‐contacting catheters. Notably, the PMPC coating with 10% PMPC performs better, as indicated by both hemolysis ratios and coagulation results, in accordance with its superior hydrophilicity and denser chain packing.

### 2.4 Outstanding Compatibility with Long, Narrow, and Complex‐Shaped Catheters

**Figure 4:**
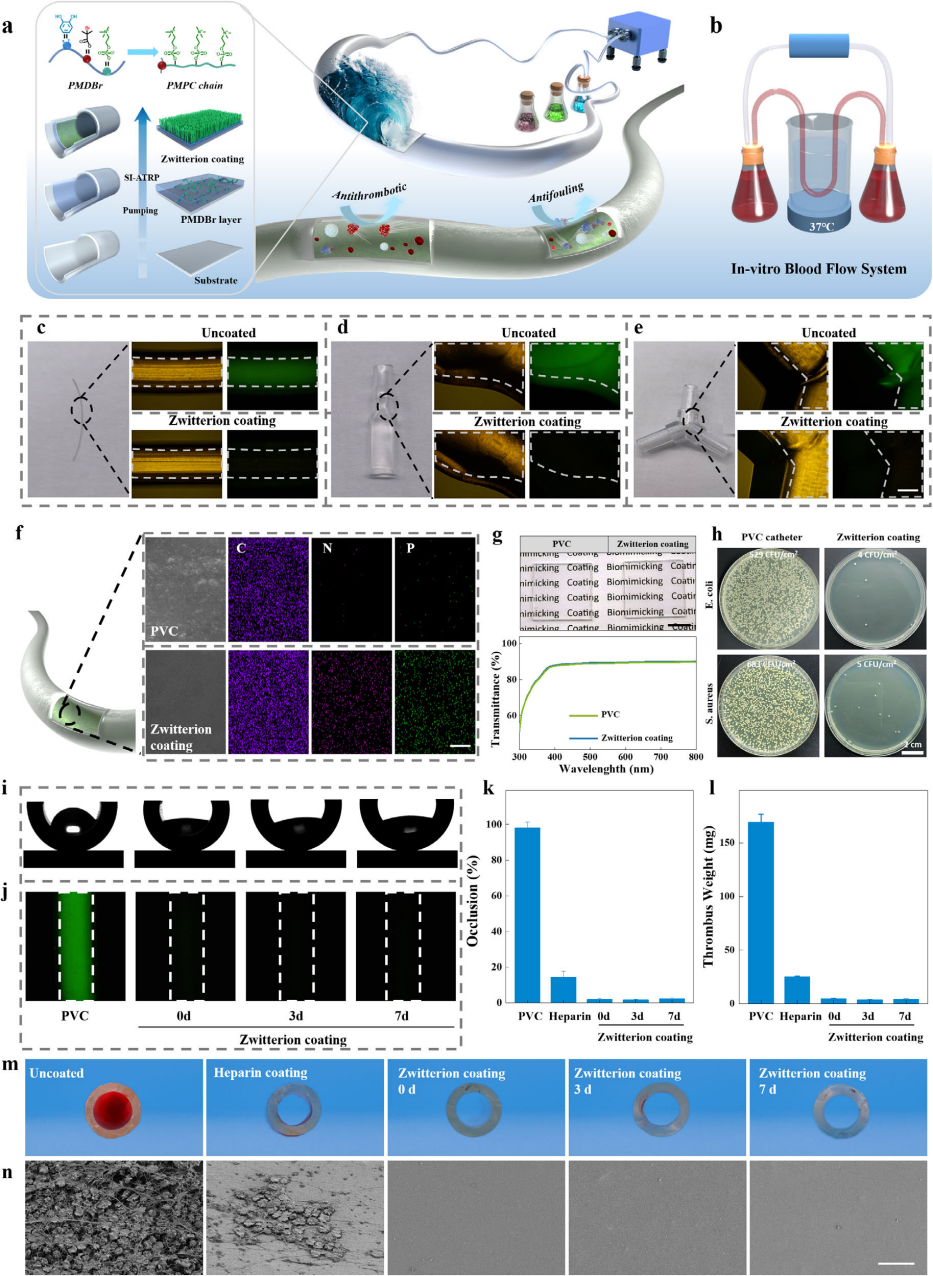
A catheter‐compatible approach to constructing anti‐thrombus and transparent inner walls. a) Schematic presentation for the preparation of the zwitterion coating in medical catheters, including pumping into subsequently catheters the solution of the copolymer of MPC, DMA, and MBr (wet‐adhesive initiator‐bearing polymer) and that of the MPC monomer and catalyst. b) Schematic presentation for the in vitro blood flow system to test the anticoagulant properties of catheters. c–e) Optical images (left), bright field images(middle), and fluorescence microscopic images (right) of tubes, reducers, and tee crosses with and without the zwitterion coatings, after incubated with FITC‐BSA. The scale bar is 1 mm. f) SEM images and EDS mapping of PVC catheter with and without zwitterion coatings. The scale bar is 3 µm. g) Optical images (up) and optical transmission spectra (down) of PVC substrate with and without the zwitterion coatings. The scale bar is 1 mm. h) Optical images of *E. coli* and *S. aureus* adhesion on the PVC catheter (right) and zwitterion‐coated catheter (left). i) Static water contact angle of the zwitterion‐coated catheter after circulation in SPSS for 0, 3, and 7 days. j) Protein adsorption resistance of the zwitterion‐coated catheter after circulation in SPSS for 0, 3, and 7 days, BSA‐FITC was selected as the model protein to provide a fluorescent signal. k) Occlusion rate and l) thrombus weight of the pristine, heparin‐coated and the zwitterion‐coated PVC catheters. m) The optical images for cross sections, after being tested by the in vitro blood flow system, for the pristine, heparin‐coated and the zwitterion‐coated PVC catheters. n) SEM images, after being tested by the in vitro blood flow system, for the pristine, heparin‐coated and the zwitterion‐coated PVC catheters. The scale bar is 20 µm.

Given all the results presented above, the PMPC coatings with 10% MBr perform better in antifouling, cell compatibility, and anticoagulation than those with 5% MBr. Therefore, from now on, we employ the PMDBr with 10% MBr to develop a zwitterion coating approach to modifying medical catheters across diverse substrates, addressing cost‐inefficiency limitations and poor catheter compatibility for previous zwitterion graft‐from methods. In detail, we successively pumped the aqueous solution of the wet‐adhesive initiator‐bearing polymer and the aqueous solution consisting of the compounds (MPC monomer, CuBr, CuBr_2_, and tris(2‐pyridylmethyl)amine (TPMA)) for growing the zwitterionic polymers through the catheters at a 16 µL min^−1^ for 10 and 45 min, respectively, with following the typical process flow (**Figure** **4**a).

To evaluate the catheter compatibility of this approach, we applied the procedure to modify various catheter components, including tubes, reducers, and tee crosses. The modified catheters were then exposed to FITC‐BSA buffer to assess the spatial uniformity of the coating and antifouling properties. No fluorescence signal was observed on any region of all PMPC‐coated parts, whereas a strong fluorescence signal could be detected on all the uncoated controls (Figure 4c–e). This result indicates that the zwitterion coating can be uniformly applied to the catheters with long and complex geometries. This conclusion was further supported by the smooth surface morphology and the significantly increased N and P content observed for the inner walls of the modified catheters by the SEM and energy dispersive spectrometer (EDS) analyses (Figure 4f).

### 2.5 Transparent, Antibacterial, Durable, and Anticoagulant Catheters

In addition, unlike most current dopa‐assisted coatings, this zwitterion coating has good transparency due to its intrinsic colorlessness and nanosized thickness, which is crucial for accurate diagnosis and treatment (Figure 4g). Through the coated and pristine PVC films, the words on the paper underneath can be seen clearly without any difference in clarity. In addition, almost no difference could be detected in the transmittance within the visible wavelength range (390 – 800 nm).

For assessing the antibacterial property of the zwitterion coating, *E. coli* and *S. aureus* were cultured in the coated catheters for 18 h. After removing unbound bacteria, the adhered bacteria were detached and further cultured to evaluate the coatings' bacterial adhesion resistance.^[^ ^66^ ^]^ Severe bacterial contamination was observed in the pristine catheter, with colony densities of 529 CFU cm^−2^ for *E. coli* and 683 CFU cm^−2^ for *S. aureus* (Figure 4h). In contrast, only a few bacteria were observed for the coated catheters, indicating its significant inhibition of bacterial adhesion and growth, with colony densities reduced to 4 CFU cm^−2^ for *E. coli* and 5 CFU cm^−2^ for *S. aureus*. These results indicate a reduction in bacterial adhesion of 99.2% for *E. coli* and 99.3% for *S. aureus*, highlighting the potential of these zwitterion‐coated catheters in preventing infections in medical applications.

The durability of zwitterion coatings is crucial for the practical application of the zwitterion grafting approach on blood‐contact catheters. The zwitterion coating remained unchanged in the surface morphology after being exposed to a continuous flow of stroke‐physiological saline solution (SPSS) for 7 days (Figure S14, Supporting Information). The elemental analysis results further confirmed the retention of the zwitterion coating after 7‐day catheter circulation (Figures S15 and S16, Supporting Information). Specifically, the C content on the surface of the uncoated PVC catheter decreased by 12.8%, likely due to surface degradation or contamination.^[^ ^67^, ^68^ ^]^ In contrast, the C, P, and N contents on the zwitterion coatings surface remained consistent with the initial values, confirming the coating's durability. The durability of the zwitterion coatings was also evidenced by the constant water contact angle during the catheter circulation (Figure 4i). Additionally, we testified the biofouling of the SPSS‐treated pristine and zwitterion‐coated catheters by exposing both to the FITC‐BSA protein solution. A strong fluorescence signal was observed on the inner walls of the uncoated PVC catheter, whereas no fluorescence signal was detected on those of the zwitterion‐coated catheter (Figure 4j). These results collectively demonstrate the durability of the zwitterion coatings, which should stem from the synergistic action of side groups, as revealed by the QCM experiment.

To further evaluate the potential of this coating in real‐world scenarios, we employed an in vitro blood circulation model (Figure 4b) that closely simulated the in vivo conditions. This mode was used to test the anticoagulant properties of the zwitterion‐coated catheter under dynamic conditions, with both a pristine catheter and a heparin‐coated catheter serving as controls. In contrast to a distinctive thrombus formation observed with the pristine catheter, only a few thrombi were detected on the inner walls of the heparin‐coated catheter after 3 h of blood circulation (Figure 4m). It indicates the good anticoagulation performance of the heparin coating, which aligns with the previous reports. However, almost no thrombus formation was observed inside the zwitterion‐coated catheter, demonstrating superior anticoagulant properties. Notably, the zwitterion‐coated catheter could maintain excellent thrombus inhibition after a 7‐day SPSS circulation, further verifying this coating's durability. To quantitatively analyze the occlusion, we calculated the area percentages of thrombus in the sections of the three catheters (Figure 4k). The results indicated its occlusion is negligible and does not worsen during the SPSS circulation. In addition, the quantitative analysis of thrombus mass gave a similar result (Figure 4l). Both measurements indicated that the zwitterion coating reduced the thrombus formation by ≈98%, whereas the heparin coating reduced it by only ≈85%, indicating that the former is significantly more effective than the latter in terms of anticoagulant performance. As observed under SEM (Figure 4n), unlike the heparin‐coated catheters, no clots were found on the inner surface of the zwitterion‐coated catheter even after 7 days of SPSS circulation. All the above results support that the zwitterion coating outperforms the heparin coating in anticoagulant properties, which should be attributed to the high density of grafted polymer chains achieved through this zwitterion graft‐from approach.

### 2.6 Zwitterion‐Coated Catheter with Selective and Efficient Cell Capture from Blood

To date, none of the existing antifouling coating approaches have demonstrated the ability to incorporate biomolecules into catheter coatings, thereby enabling their specific biofunction besides their anticoagulant properties. However, integrating biofunctional elements into anticoagulant catheters is essential, especially given the wide range of clinical challenges and complications associated with blood‐contacting medical devices. Previous trials using other techniques have shown that incorporating biomolecules into catheters can enhance anticoagulation, provide anti‐infection properties, or facilitate in vivo or ex vivo capture/remove of tumor cells and pathogens.^[^ ^69^, ^70^, ^71^, ^72^ ^]^

**Figure 5:**
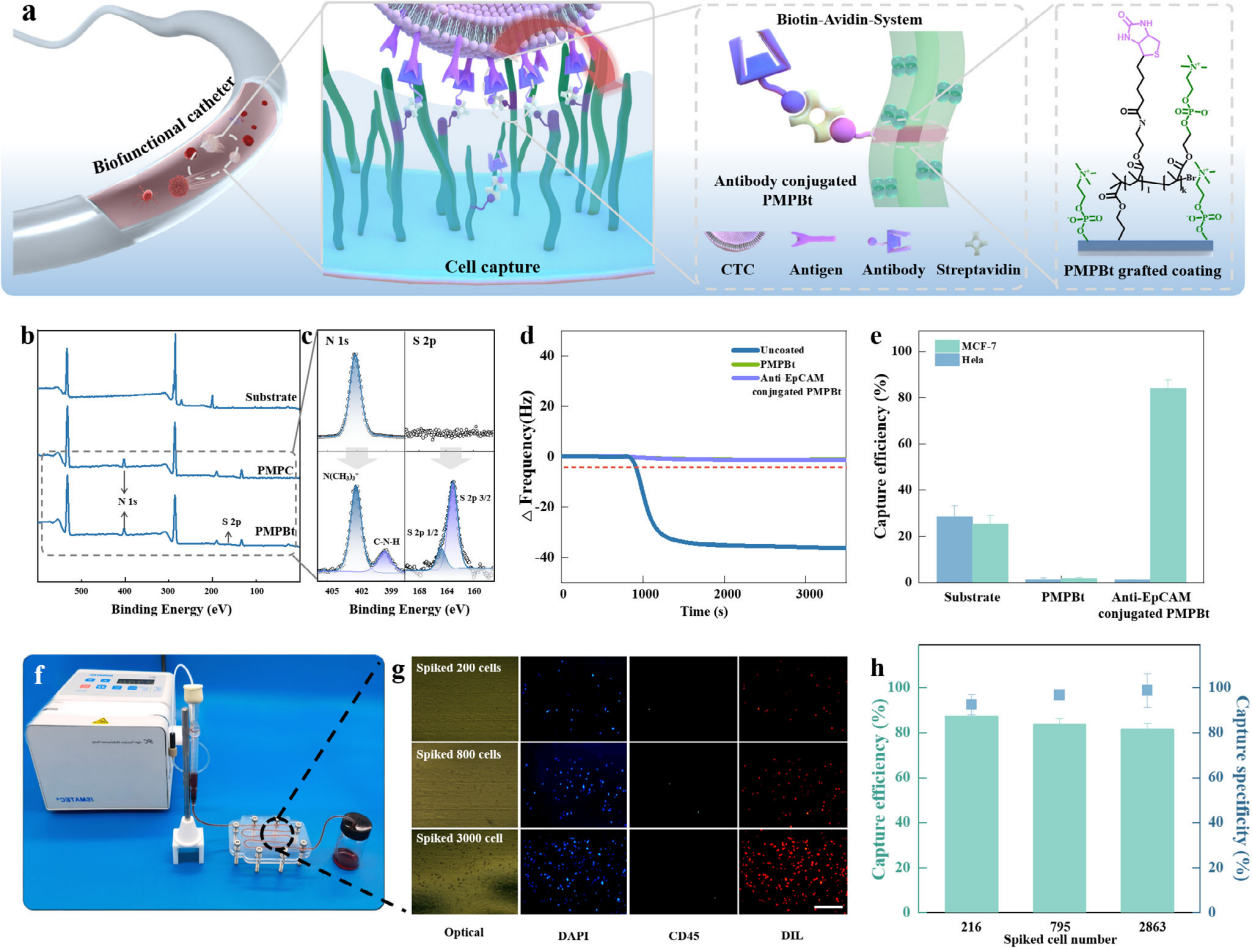
A biofunctionalized catheter capable of removing selectively and efficiently tumor cells from blood. a) Schematic presentation for the circulating‐tumor‐cell (CTC) capturing catheters, in which inner walls are grafted by the PMPBt chains with anti‐EpCAM antibodies. b) XPS survey spectra of the pristine, the PMPC‐grafted coating, and the copolymer of MPC and biotin‐functionalized methacrylate (MBt) grafted catheters. c) High‐resolution XPS spectra of N 1s (left) and S 2p spectra (right) for the PMPC‐grafted coating (top), and the PMPBt‐grafted coating (bottom). d) In situ monitoring of the frequency change (Δf) by QCM for the FBS adsorption of the pristine, the PMPC‐grafted, and the PMPBt‐grafted gold QCM crystals. e) MCF‐7 and Hela cell capture efficiency of pristine, PMPBt‐coated, and anti‐EpCAM conjugated PMPBt‐coated with PVC substrates. The bars represent the mean ± SD (*n* = 3). f) Optical images of the CTC capture microfluid device. g) Fluorescence image of MCF‐7 cells captured on the anti‐EpCAM conjugated PMPBt‐coated PVC catheters. The scale bar is 150 µm. h) The efficiency and specificity of the MCF‐7 cell capture by biofunctionalized catheters. The bars represent the mean ± SD (*n* = 3).

To address this issue, we grafted copolymers of MPC and the biotin‐functionalized methacrylate (MBt) (PMPBt) instead of PMPC chains through the 2‐bromoisobutyrate groups presented on the surface by pumping in a solution where MBt and MPC make up 5% and 95% of the overall monomer, respectively. The mole percentage of MBt in the whole monomer is controlled at 5% to retain the excellent antifouling performance of this coating. Subsequently, we conjugated the biotinylated anti‐EpCAM antibody onto this zwitterion coating using streptavidin as an intermedium (**Figure** **5**a). We thus constructed a specific interaction toward the EpCAM‐presented circulating‐tumor‐cell (CTCs) on a nonspecific binding resisting background. This way, the anti‐EpCAM molecules presented on polymer chains formed a CTC‐selective interaction network on the inner wall of tubes. The XPS spectra of the PMPBt coating, being different from that of the PMPC coating (Figure 5b), additionally present S 2p (162.9 eV) and N 1s (399.2 eV) peaks arising from biotins, indicating the successful introduction of MBt monomer into the grafted hydrophilic polymer chains. In addition, the binding energy of the quaternary ammonium N 1s of MPC (402.6 eV) is higher than that of ‐N‐H N 1s of MBt (399.6 eV), making it possible to calculate the actual composition of MBt based on the areas of two peaks (Figure 5c). The results show that the composition of MBt is ≈5%, which is well in accordance with that at feed.

We further checked the protein fouling of the PMPBt coating and the antibody‐conjugated PMPBt coating, including the PMPC coating as a control. Being similar to the PMPC coating, both presented strong resistance to the nonspecific fetal bovine serum (FBS) binding, indicating that the MBt incorporation and the antibody grafting did not compromise the nonspecific interaction resistance of coatings (Figure 5d). To evaluate the specificity and capture efficiency of this interaction network combined coating, we cultured the EpCAM‐positive Michigan Cancer Foundation‐7 cells (MCF‐7 cells) and the EpCAM‐negative Henrietta Lacks cells (Hela cells) on the pristine, the PMPBt coated and the antibody‐conjugated PMPBt coated substrates (Figure 5e and Figure S21, Supporting Information). Both cells (25.4% for MCF‐7 and 28.3% for Hela) could attach to the pristine substrate due to the nonspecific nature of its cell‐substrate interaction. In contrast, neither of these cells (1.46% for MCF‐7 and 1.88% for Hela) could attach to the PMPBt coating due to its strong resistance to nonspecific cell binding. On the other hand, only MCF‐7 cells (85.14% for MCF‐7 and 1.07% for Hela) could attach to the antibody‐conjugated zwitterion coating. We attributed its excellent cell selectivity to the collaborative effect of specific interaction and nonspecific interaction resistance. Furthermore, the cell capture efficiency of the MCF‐7 cells of the antibody‐conjugated PMPBt coating was almost three times that of the pristine substrate, indicating the strong affinity of this specific interaction network.

We then applied the aforementioned procedure to deposit the antibody‐conjugated coating onto a PVC catheter (40 cm in length and 0.3 cm in diameter) and investigated the feasibility of this dual‐functional catheter for dynamically isolating CTCs (Figure 5f). To prepare the simulated blood samples, we spiked the DIL‐prestained MCF‐7 cells into the simulated blood at concentrations of 216, 795, and 2863 cells mL^−1^. The simulated blood sample was infused into the catheter at 16.7 µL min^−1^. To distinguish CTCs from nonspecifically bound leukocytes, we used the FITC‐labeled anti‐CD45 (green) and DAPI (blue) to stain further all the cells trapped in the tube. Almost all the cells trapped in the tube are CTC cells (DAPI+/DIL−/CD45‐), except for a very few leukocytes (DAPI+/DIL−/CD45+) (Figure 5g). The cell capture efficiency for all three samples exceeded 80%, with a significant increase in the efficiency as cell density decreased. The cell capture efficiency of this tube increased from 81.6% to 87.5%, with the cell density decreasing from 2836 to 200 cells mL^−1^ (Figure 5h). This unique dependence of cell capture efficiency on the cell concentration makes this tube particularly useful to isolate CTCs from blood, as the number of CTCs in the blood is extremely small. Moreover, its cell capture selectivity was as high as 97%, without dependence on the cell densities of tumor cells in the simulated blood. This antibody‐conjugated coating endows this tube with high cell capture efficiency and selectivity, thus offering an opportunity to capture/remove CTCs from blood besides resisting the nonspecific binding of blood cells. Besides antibodies, we could also conjugate other biomolecules, e.g., anticoagulant groups and antibacterial factors, to further enhance the biocompatibility of catheters. Therefore, we envision that this zwitterion graft‐from coating strategy offers a versatile and general approach to engineering the inner lumen of medical catheters.

## 3 Conclusion

We have demonstrated a cost‐efficient, mechanically viable, and universal strategy for grafting superhydrophilic and natural zwitterionic polymers from narrow, long, and complex catheters. This approach, utilizing a newly synthesized wet‐adhesive initiator‐bearing polymer, enables rapid and robust assembly on the catheter inner walls through synergistic side group action in aqueous environments, efficiently addressing the slow modification and weak substrate interaction of other supermolecular methods. It is compatible with a wide range of substrates, including metals, polymers, and oxides, and demonstrates exceptional adaptability to catheters and joints of various lengths and shapes. After the grafting of zwitterionic polymers, the resulting coatings exhibit exceptional antifouling and antithrombotic properties, achieving near‐zero protein, blood cell, and bacteria fouling with wide pH adaptability. In addition, the zwitterion‐coated catheters reduced the thrombus formation by ≈98%, much superior to the heparin coating (≈85%). Furthermore, the coatings demonstrate excellent stability and durability under simulated bloodstream, ensuring long‐term functionality. Importantly, this strategy also enables the creation of a highly selective biointeraction network on catheter surfaces, facilitating the precise and efficient capture of circulating tumor cells from the bloodstream. Overall, this zwitterion graft‐to approach provides a cost‐efficient and robust solution for modifying medical catheters with complex geometries, combining rapid modification, mechanical durability, outstanding anticoagulant performance, and customizable bio‐functionality to meet diverse clinical needs. We envision that this method is not limited to medical catheters; its rapid, activation‐free, and substrate‐independent characteristics make it easily adaptable to a wide range of medical devices with complex surfaces exposed to challenging biological environments.

## 4 Experimental Section

### Materials and Reagents

Dopamine hydrochloride, methacrylic anhydride, sodium bicarbonate (NaHCO_3_), sodium borate decahydrate (Na_2_B_4_O_7_·10H_2_O), tris(2‐pyridylmethyl)amine (TPMA), azobisisobutyronitrile (AIBN), CuBr_2_, and CuBr were purchased from Sigma‐Aldrich and used without further purification. Glutaraldehyde (2.5%) and 60U heparin were purchased from Aladdin Inc. 2‐(2‐bromoisobutyryl) ethyl methacrylamide (MBr) and 2‐methacryloyloxyethyl phosphorylcholine (MPC) were purchased from Yuhao Chemical. Solvents like dimethylformamide (DMF), hexane, tetrahydrofuran (THF), and dimethyl sulfoxide (DMSO) were purchased from Greagent. All the chemical agents were of analytical grade. The Luria‐Bertani (LB) solid medium (1 L) contained yeast extract (5 g), sodium chloride (10 g), agar (15 g), and tryptone (10 g). The LB liquid medium (1 L) contained yeast extract (5 g), sodium chloride (10 g), and tryptone (10 g). *E. coli* (ATCC 25 922), and *S. aureus* (ATCC 25 923) were obtained from Beijing Beina Biotechnology Company, China.

Streptavidin, bovine serum albumin (BSA), human fibrinogen (HFg), fluorescein‐isothiocyanate‐labeled bovine serum albumin (FITC‐BSA), and fluorescein isothiocyanate‐labeled human fibrinogen (FITC‐HFg) were purchased from Thermo Fisher Scientific Inc. Biotinylated anti‐EpCAM was purchased from R&D system. The PDMS plates were prepared following procedures previously reported.^[^ ^73^ ^]^ The gold plates were prepared by electron beam sputtering using the previously reported approach.^[^ ^74^ ^]^ The SiO_2_, PVC, and other plates were purchased from South China Xiangcheng Technology Inc. The catheters were purchased from Guangzhou Saituo Technology Inc.

The henrietta lacks cells (Hela), mouse embryonic fibroblast cell line (NIH3T3 cells), and michigan cancer foundation‐7 cell (MCF‐7) lines were provided by Stem Cell Bank, Chinese Academy of Sciences. The fresh Kunming mice whole blood (EDTA, 1.5 mg mL^−1^) was legally acquired from Berseebio Technology Inc. following the ethics standards. High glucose Dulbecco's modified Eagle's medium (DMEM), Minimum essential medium (MEM), GlutaMAXTM supplement, fetal bovine serum (FBS), nonessential amino acids, bovine calf serum, penicillin‐streptomycin, sodium pyruvate solution, and other additives for cell culture were purchased from Corning Co. (Manassas). 1,1′‐dioctadecyl‐3,3,3′,3′‐tetramethylindocarbocyanine perchlorate (red DIL stain), 3,3′‐dioctadecyloxacarbocyanine perchlorate (green DIO cell stain), 4′,6‐diamidino‐2‐phenylindole (blue DAPI cell staint), Calcein Acetoxymethyl Ester/Propidium Iodide (calcein‐AM/PI) double staining kit were purchased from Beyotime Biotechnology Inc. Anti‐CD45 conjugated with fluorescein isothiocyanate (FITC‐labeled anti‐CD45) were purchased from BioLegend.

### Synthesis

N‐(3,4‐Dihydroxyphenyl)ethyl methacrylamide (DMA) and biotin‐functionalized methacrylate (MBt) were synthesized following procedures previously reported.^[^ ^75^, ^76^ ^]^ The ^1^H NMR of the synthesized product is given in Figure S1 (Supporting Information).

The copolymers of MPC, DMA, and MBr were synthesized by radical copolymerization with AIBN as the initiator. Typically, DMA (128 mg, 0.58 mmol), MPC (479 mg, 1.624 mmol), and MBr (32 mg, 0.116 mmol) were added into a 100 mL Schlenk‐type flask under a nitrogen atmosphere. A solution of AIBN (19 mg, 0.116 mmol) in DMF/H_2_O (v/v, 4/1, 4 mL) was injected. The flask was degassed with three freeze‐pump‐thaw cycles and immersed in an oil bath at 75 °C for 3 h. The polymerization was quenched by immersion of a flask in liquid nitrogen. The turbid mixtures were then dialyzed against deionized water for 2 days using a Spectra/Por regenerated resin membrane with a molecular cutoff (MWCO = 3500). The whole process was performed at 20 °C. Finally, the polymer was freeze‐dried under vacuum for 24 h. FTIR (KBr, cm^−1^): 3442 (phenolic hydroxyl), 3029 (benzene ring CH stretching), 2952 (CH_2_ stretching), 1725 (C═O stretching), 1625 (benzene ring C═C stretching), 1490 (C (O)–N stretching), 1226 (P═O stretching), 1072 (P─O─C stretching), 957 (N^+^─C stretching).

### Characterization Techniques

^1^H NMR spectra were recorded on a Bruker AVANCE NEO 600 (600 MHz) spectrometer in a 1:1 (v/v) mixture of DMSO‐d6 and D_2_O, and chemical shifts were reported as values (ppm) relative to the internal Me_4_Si. These spectra were collected as 128 transients with a delay time of 10 s.

The particle size and distribution in solution were recorded by laser diffractometry using a Nano Size Particle Analyzer (Zetasizer Nano ZS90, Malvern) in the range of 0.2 nm to 5.0 µm under the following conditions: particle refractive index of 1.59, particle absorption coefficient of 0.01, solvent refractive index of 1.41, viscosity of 2.1 cP, and temperature of 25 °C. Thirteen measurement cycles of 10 s each were performed, and the average was calculated using Zetasizer Software.

The FTIR spectra of the synthesized polymer materials were recorded on an instrument (Nicolet iS10, Thermo Fisher Scientific) in the range of 400–4000 cm^−1^ with a resolution of 4 cm^−1^. The samples were thoroughly grounded, mixed with KBr, and pressed into thin sheets for the FTIR measurement.

The chemical compositions of the samples were recorded on an X‐ray photoelectron spectroscopy (Thermo K‐Alpha) using Al Kα radiation, and the binding energies were calibrated by referring to the C 1s line at 284.8 eV from adventitious carbon. A spot size of 500 µm, an energy step size of 0.1 eV, and the standard lens mode were used for the XPS measurements. High‐resolution scans were also recorded to calculate the chemical compositions.

The static contact angles of water on various surfaces were recorded using a contact‐angle measurement system (Attension Theta Flex, Biolin Scientific). The deionized water with a drop volume of 4 µL was used as the wetting liquid. Each sample was measured thrice to calculate the average contact angle value.

The Atomic Force Microscope (AFM) images of substrates were recorded using a Bruker Multimode 8 in tapping mode. The in situ liquid‐phase AFM images of the substrates in water were recorded using a Bruker Dimension FastScan Bio (using SNL‐10 Bruker probes, the force constant of 0.084 N/m, and resonant frequency of 65 kHz) in contact mode. Scanning rates were optimized at 5 µm s^−1^ with a scan resolution of 256 samples per line. Then, the AFM images and roughness parameters were identified using the NanoScope Analysis 1.7 software.

Scanning electron microscopy (SEM, Hitachi, Regulus 8100, operating voltage: 3 kV) was used to observe the morphology of the sample. Element mapping of the channels was carried out by energy‐dispersive spectroscopy (EDS, Smart EDX, operating voltage: 15 kV, energy spectrum analysis working distance: 8.5 mm) on the SEM instrument.

The optical transmittance performances of the coated substrates were tested using a UV–vis spectrophotometer (UV2600i, PerkinElmer) with air as the baseline reference. All the UV–vis spectra were recorded over a wavelength range of 300 to 800 nm.

QCM measurements were performed at 25 °C on a Q‐Sense Analyzer system (QSense Analyzer, Biolin Scientific) with a four‐channel flow cell to monitor the change on the surface in situ. The QSX 301 sensor crystal (Biolin Scientific) was placed in the measurement chamber for each measurement. The solution was continuously delivered to the measurement chamber at a flow rate of 30 µL min^−1^ under pumping by an Ismatec ISM597D pump. Upon interaction of the matter in solution with the substrate on the sensor crystal, the changes in the resonance frequency of the sensor (Δf), which is related to the attached mass, were recorded at a resolution of less than 1 s. If necessary, the pump was stopped for a few seconds to change the sample solutions without disturbing the QCM‐D signal. The resonance frequencies were measured simultaneously at 5 MHz and its five harmonics (15, 25, 35, 45, 55, and 65 MHz). If not stated otherwise, changes in the frequency of the third overtone (*n* = 3;, i.e., 15 MHz) were presented. Computational modeling of QCM‐D signals was carried out in order to determine the mass and thickness of the PMDBr layer on a gold chip using Qsense Dfind software provided by Biolin Scientific. The software offers computational methods to determine the mass adsorbed on the sensor's surface, including the Sauerbrey equation, calculated from the frequency shift and viscoelastic modeling, based on the Voigt‐Voinova model.^[^ ^77^ ^]^

### Zwitterionic Polymer Grafting Approach for Substrates

The substrate was cleaned by ultrasonication in deionized water and ethanol for 5 min each. Then, the freshly cleaned substrates were immersed in the 1.5 mg mL^−1^ solution of PMDBr (DMSO/H_2_O, v/v, 1/1, 5 mL) for 10 min under shaking on an orbital shaker (MIX‐1500, MILAB). The modified substrate was then rinsed thoroughly with distilled water and dried under a nitrogen gas flow, followed by polymer grafting, as shown below.

The zwitterionic polymers were then grafted from the PMDBr‐coated substrates by Atom Transfer Radical Polymerization (ATRP) to form an antifouling coating. A typical operation was shown below: the PMDBr‐coated substrates were placed in a reactor under a nitrogen atmosphere. Subsequently, CuBr (1.29 mg, 2 mmol), CuBr_2_ (3.01 mg, 3 mmol), TPMA (13.1 mg, 10 mmol), and MPC monomer (531.48 mg, 400 mmol) were added to the reactor. A solution of DMSO/H_2_O (v/v, 1/1, 4.5 mL) was then injected. The reactor was immersed in an oil bath at 50 °C for 45 min to obtain PMPC‐grafted substrates.

### Biofunctionalized Zwitterionic Polymer Grafting Approach for Substrates

The PMPBt was grafted from the PMDBr‐coated substrates by ATRP to form a biofunctionalized PMPBt grafted coating. A typical operation was shown below: the PMDBr‐coated substrates were placed in a reactor under a nitrogen atmosphere. Subsequently, CuBr (1.29 mg, 2 mmol), CuBr_2_ (3.01 mg, 3 mmol), TPMA (13.1 mg, 10 mmol), MPC monomer (531.48 mg, 400 mmol), and MBt monomer (33.59 mg, 21 mmol) were added to the reactor. A solution of DMSO/H_2_O (v/v, 1/1, 4.5 mL) was then injected. The reactor was immersed in an oil bath at 50 °C for 55 min to obtain PMPBt‐grafted substrates.

After the PMPBt‐grafted substrates were immersed in PBS buffer for 1 h, a 100 µL streptavidin solution (50 µg mL^−1^ in PBS buffer) was spread on the PMPBt‐grafted surfaces and then left to incubate at 25 °C for 50 min, followed by PBS buffer rinsing to remove excess streptavidin. Then, a 100 µL biotinylated anti‐EpCAM antibody solution (10 µg mL^−1^ in PBS buffer) was spread on the streptavidin‐modified PMPBt surface, and the substrates were incubated at 37 °C for 3 h. Finally, the substrates were rinsed with PBS buffer to remove excess antibodies.

### Biofunctionalized Zwitterionic Polymer Grafting Approach for Catheters

A 1.5 mg mL^−1^ PMDBr solution (DMSO/H_2_O, v/v, 1/1) was pumped into a catheter by a peristaltic pump (ISM935C, ISMATEC) for 10 min at a flow rate of 16 µL min^−1^ and 25 °C, followed by the distilled water rinsing to remove the unbound PMDBr. Next, a solution containing MPC monomer (531.48 mg, 400 mmol), MBt monomer (33.59 mg, 21 mmol), CuBr (1.29 mg, 2 mmol), CuBr_2_ (3.01 mg, 3 mmol), and TPMA (13.1 mg, 10 mmol) in DMSO/H_2_O (v/v, 1/1, 4.5 mL) was pumped into the catheter over 55 min at a flow rate of 16 µL min^−1^ and 50 °C, followed by thorough distilled water rinsing. Then, a streptavidin solution (50 µg mL^−1^ in PBS buffer) was pumped through the catheter for 50 min at a flow rate of 16 µL min^−1^ and 25 °C, followed by 10 min of PBS buffer rinsing. Finally, a biotinylated anti‐EpCAM antibody solution (10 µg mL^−1^ in PBS buffer) was pumped through the catheter for 180 min to conjugate the antibody onto the grafted polymer chains.

### Evaluation of Antifouling and Biocompatibility of Substrates—QCM for Protein Adsorption Assay

The protein adsorptions of substrates to various proteins, including BSA, HFg, and FBS, were assayed by QCM. BSA and HFg were dissolved in the PBS buffer at 1 mg mL^−1^ to prepare their protein solution. The FBS was diluted tenfold in PBS buffer (v/v, 1:10) for the protein adsorption assay. The pH of the HFg and FBS was adjusted using NaOH and HCl solution before QCM‐D tests.

### Evaluation of Antifouling and Biocompatibility of Substrates—Fluorescence‐Labeled Protein Adsorption Assay

FITC‐BSA and FITC‐HFg were used to visualize the nonspecific protein adsorption of substrates. The FITC‐BSA (1 mg mL^−1^ in PBS buffer) and FITC‐HFg (1 mg mL^−1^ in PBS buffer) solutions were applied to the substrate surface and incubated at 25 °C for 1 h, followed by thorough PBS buffer washing. All the fluorescence images were taken under a fluorescence microscope (CKX‐53, Olympus), and all the above processes were performed in darkness.

### Evaluation of Antifouling and Biocompatibility of Substrates—Blood Platelet, Erythrocyte, and Leukocyte Adhesion

The platelet, erythrocyte, and leukocyte were prepared by centrifuge sorting. One mililiter fresh Kunming mice whole blood was centrifuged using a low‐speed centrifuge (TDZ4A‐WS, CENCE) at 1500 rpm for 15 min. The platelet‐rich plasma (PRP) portion was collected carefully with a pipet without disturbing the buffy coat. Then, the PRP was further centrifuged at 3500 rpm for 5 min to isolate platelets, and the sediments (platelets) were collected, followed by resuspension in 1 mL PBS buffer.

After being diluted two‐fold in PBS buffer, 2 mL of diluted blood sample was centrifuged at 1200 rpm for 5 min to isolate erythrocytes. The sediments were collected, washed, centrifuged, and resuspended in 1 mL of PBS.

Two milliliters diluted blood sample was carefully layered on a 1.5 mL lymphocyte separation medium (Ficoll‐Paque PLUS, Amersham Biosciences) in a 15 mL centrifuge tube. The centrifuge tube was then centrifuged at 1580 rpm for 20 min to isolate lymphocytes. The second layer was carefully collected with a pipe, washed, centrifuged, and resuspended in 1 mL of PBS buffer.

For evaluating the cell adhesion of various coatings, 200 µL of isolated blood cell sample was allocated onto the substrate surface and incubated for 120 min at 37 °C. After seeding the blood cells, the substrates were washed gently three times with the assay buffer diluted ten‐fold with distilled water. The substrates with platelet were incubated in 5 mm DIO in PBS buffer for 30 min at 37 °C and 5% CO_2_. Those with erythrocytes were treated similarly with 5 mm DIL. Different from the above samples, the substrates with leukocytes were treated first with 4% paraformaldehyde solution before cell staining. These samples were permeabilized with 0.1% Triton X‐100 for 5 min and incubated in 1 mm DAPI in PBS buffer for 5 min at 37 °C and 5% CO_2_. All the substrates were rinsed thoroughly with assay buffer before being observed under a fluorescence microscope (CKX‐53, Olympus). The adherent cell density was quantified from fluorescent images using ImageJ software.

### Evaluation of Antifouling and Biocompatibility of Substrates—Cytotoxicity Assay

Before cell experiments, all coating and substrates were immersed in PBS for 2 h and sterilized in a 75% ethanol solution for 30 min. NIH3T3 cells were used as the model cells to investigate the cytocompatibility of coatings. The NIH3T3 cells were cultured in DMEM supplemented with 10% bovine calf serum, 1% GlutaMAX supplement, 1% sodium pyruvate solution, 1% nonessential amino acids, and 1% penicillin‐streptomycin. All cells were cultured at 37 °C and under 5% CO₂. Cytotoxicity assessments were conducted following the ISO10993‐5 standard. Extracts were prepared by incubating the substrate in a DMEM medium at 37 °C and under 5% CO_2_ for 48 h at a surface area to extraction medium ratio of 5 cm^2^ mL^−1^. Cells were seeded in 96‐well plates at a density of 1 × 10^5^ cells/200 µL and cultured for 12 h. Then, the culture medium in wells was replaced with 200 µL of the extract, with the pristine culture medium serving as the negative control. Calcein‐AM/PI double staining kit after further culturing for 12, 24, and 72 h. After three washes with the assay buffer (diluted tenfold in the distilled water), the substrates were incubated in the serum‐free DMEM containing 2 mm calcein acetoxymethyl ester and 1.5 mm propidium iodide for 30 min at 37 °C and under 5% CO_2_. The samples were then rinsed thrice with the diluted assay buffer and observed under a fluorescence microscope (CKX‐53, Olympus). Cell viability analyses were carried out following the standard protocol.

### Evaluation of Antifouling and Biocompatibility of Substrates—Hemolysis Ratio Assay

Following the method mentioned above, the substrate extracts were prepared using PBS buffer as the extraction medium. 0.2 mL of erythrocyte dispersion was added into 0.8 mL extract to prepare the sample for measuring the hemolysis ratio of substrates (with PBS buffer as a negative control and distilled water as a positive control). After being incubated at 37 °C for 2 h, the erythrocyte‐containing extracts were centrifuged at 2400 rpm for 4 min to remove the remaining erythrocytes. The absorbance of the supernatant at 542 nm was then recorded using a UV–vis spectrometer (UV2600i, PerkinElmer). Each sample was measured thrice to give an averaged hemolysis ratio value. The hemolysis ratio was calculated using equation (1), as shown below.

(1) $$\mathrm{Hemolysis}\;\mathrm{ratio}=\left(\left(A_{T}-A_{N}\right)/\left(A_{p}-A_{N}\right)\right)\times 100\%$$

where A_T_ is the absorbance of the test sample, and A_N_ and A_P_ are the absorbance of the negative and positive control, respectively.

### Evaluation of Antifouling and Biocompatibility of Substrates—Anticoagulation Assay

Hundred micorliters Kunming mice whole blood and 15 µL CaCl_2_ solution (0.2 mol L^−1^ in saline solution) were added successively onto the surface of coatings. After incubated at 37 °C for 60 min, the samples were immersed in 5 mL of distilled water for 5 min.

### Evaluation of Antifouling and Biocompatibility of Catheters—Protein Adhesion Assay

The FITC‐BSA solution in PBS buffer (1 mg mL^−1^) was pumped into catheters and incubated for 1 h, followed by thorough washing with PBS buffer. The catheter was observed under a fluorescence microscope (CKX‐53, Olympus). All processes were performed in darkness.

### Evaluation of Antifouling and Biocompatibility of Catheters—Antibacterial Ability Test

The antibacterial experiment was conducted using *E. coli* and *S. aureus* following the ISO 22196‐2011 standard. Bacteria were cultured on a solid agar medium at 37 °C for 24 h. Individual colonies were then transferred into a liquid culture medium and incubated at 37 °C for 24 h. The bacterial suspensions were diluted to concentrations ranging from 5 × 10^6^ to 1 × 10^7^ CFU mL^−1^ using the nutrient solution. Then, 500 µL of the standardized bacterial suspension was evenly dispersed onto the sample surfaces and incubated at 37 °C for 18 h. The samples were then gently washed with PBS three times to remove loosely‐adhered bacteria. An intermittent ultrasonication treatment was applied to samples to detach the adhered bacteria, which were then collected and spread onto the solid agar medium. After incubation at 37 °C for 24 h, the bacterial growth on the plates was monitored through photography, and bacterial counts were performed using ImageJ software.

### Evaluation of Antifouling and Biocompatibility of Catheters—Stability Assessment

PMPC‐coated catheters were connected to a peristaltic pump, and a stroke‐physiological saline solution (SPSS) was circulated at a flow rate of 65 mL min^−1^ and a temperature of 37 °C for 7 days. On the 3 and 7 days, water contact angle measurements were performed, and the inner wall morphology of the catheters was measured using SEM observation. Additionally, the element distribution on the surface was measured by EDS. Furthermore, after 3 and 7 days, the catheters were immersed in FITC‐BSA solution for 1 h, followed by thorough washing with PBS buffer. The catheter was observed under a fluorescence microscope (CKX‐53, Olympus) to evaluate the stability of its antifouling performance.

### Evaluation of Antifouling and Biocompatibility of Catheters—Anti‐Thrombogenicity Test

A blood circulation device was constructed to simulate the blood circulation and evaluate the anti‐thrombogenicity properties of the modified catheters. The setup included the tested catheters, a peristaltic pump, two Erlenmeyer flasks, and two connecting tubes for the peristaltic pump and flasks.

Before the anti‐thrombogenicity test, all components were rinsed with 75% alcohol, saline solution, 60 U heparin, and saline solution, ensuring the lumen of the whole apparatus was antithrombotic. A heparin‐coated catheter was prepared as a control by immersing a catheter in a 60 U heparin solution and soaking it at 25 °C for 24 h. Whole blood from Kunming mice was used for the test, and CaCl_2_ solution (0.2 mol L^−1^ in saline) was added to the blood to counteract anticoagulants and accelerate thrombosis. Before the test, the original PVC catheter, heparin‐coated catheter, and PMPC‐coated catheter were individually connected to the circulation device, and 40 mL of whole blood from Kunming mice was injected. The blood was circulated at a flow rate of 5 mL min^−1^ for 3 h, followed by gentle washing with PBS buffer at the same flow rate. The sample was then removed from the device and placed in a drying oven at 50 °C for 12 h. After drying, the samples were weighed to calculate the thrombus weights.

To evaluate thrombus formation, the fresh samples were cut and fixed for 12 h in 2.5% (wt/vol) glutaraldehyde dissolved in a filter‐sterilized PBS buffer and then rinsed thrice with distilled water. The fixed samples were dehydrated in successive ethanol‐water mixtures with increasing ethanol concentrations of 25%, 50%, and 75% by volume for 10 min each and then twice in pure ethanol for 10 min each. The samples were dried by critical point drying and then coated with gold using a sputter coater (Hitachi, MC1000). The thrombus formed on the inner of the specimens was observed by SEM (Hitachi, Regulus 8100) at 2 kV.

### Tumor Cell Adhesion Experiments—Cell Culture Experiment

Before the cell culture experiment, all the substrates were immersed in PBS for 12 h, followed by 30 min of sterilization in a 75% ethanol solution. All the cell culture experiments were conducted at 37 °C and under 5% CO_2_. MCF‐7 cells were cultured in MEM supplemented with 10% FBS, 1% GlutaMAX, 1% sodium pyruvate solution, 1% nonessential amino acids, 1% penicillin‒streptomycin, and 0.014 mg mL^−1^ insulin. Hela cells were cultured in DMEM supplemented with 10% FBS, 1% sodium pyruvate solution, and 1% nonessential amino acids.

### Tumor Cell Adhesion Experiments—Tumor Cell Capture on Substrates

The coated and pristine PVC substrates were placed in the wells of a 24‐well plate for cell culture. Before preparing the cell suspension, the MCF‐7 and Hela cells were stained with DIO and DIL, respectively, according to the manufacturer's protocols. Then, 200 uL cell suspensions of MCF‐7 (1 × 10^5^ cells mL^−1^) and Hela cells (1 × 10^5^ cells mL^−1^) were seeded on the sample surfaces, respectively. After the cells were cultured for 2 h, the sample surfaces were washed thrice with PBS buffer and observed under a fluorescence microscope (CKX‐53, Olympus). The cell density for the cells attached to the substrate was quantified from five randomly taken fluorescent images using ImageJ software.

### Tumor Cell Adhesion Experiments—Tumor Cell Capture by Catheters

A simulated blood sample spiked with MCF‐7 cells was prepared to assess the selective interactions of the PMPBt coating on the catheter's inner walls with tumor cells in the presence of leukocytes. The details are given below. 1 mL of Kunming mice whole blood with anticoagulant EDTA was lysed at 25 °C for 5 min after adding 10 mL 1× red blood cell lysis buffer. After diluted in 20 mL of PBS buffer, the lysate was centrifuged at 1680 rpm for 5 min to facilitate leukocyte isolation. The isolated leukocytes were resuspended in a 1 mL standard medium (MEM supplemented with 10% FBS, 1% GlutaMAX, 1% sodium pyruvate solution, 1% nonessential amino acids, 1% penicillin‐streptomycin, and 0.014 mg mL^−1^ insulin) for further use.^[^ ^78^ ^]^ Before preparing the mixed cell suspension, MCF‐7 cells were stained with DIL according to the manufacturer's protocols. Then, the MCF‐7 cells (216, 795, 2863 cells) were added to the leukocyte dispersion (1 × 10^6^ cells mL^−1^) to obtain a simulated blood sample with tumor cells.

An antibody‐functionalized PMBt‐coated catheter was prepared according to the method described in Section 4.6. Two milliliters simulated blood sample was pumped into the catheter at a flow rate of 16 µL min^−1^, followed by pumping PBS buffer at a flow rate of 70 µL min^−1^ for 10 min. Then, 4% paraformaldehyde solution was pumped into the catheter for 10 min to fix the cells. The FITC‐labeled anti‐CD45 solution in PBS buffer (10 µg mL^−1^) was then pumped into the catheter (30 µL min^−1^) and incubated for 3 h to stain the leukocytes. Subsequently, 500 µg mL^−1^ DAPI solution in PBS buffer was pumped into the catheter (30 µL min^−1^) for 30 min to stain the nuclei. After rinsing by pumping PBS buffer at 200 µL min^−1^ for 20 min, the catheters were observed under a fluorescence microscope (CKX‐53, Olympus).

## Conflict of Interest

The authors declare no conflict of interest.

## Author Contributions

T.Z. and T.L. contributed equally to this work. B.Z., T.Z., and T.L. initiated the project and designed the experiments. T.Z., T.L., and Q.P. synthesized the monomers and macroinitiators and characterized these materials. T.Z., T.L., and Q.P. carried out the surface‐initiated ATRP and evaluated the antifouling and anticoagulant properties of the modified surfaces. T.Z., T.L., and S.Z. modified catheters and evaluated their anticoagulant and selective cell‐capturing performances. T.Z. wrote the manuscript draft, and B.Z., S.Z., and Z.G. revised the manuscript. All of the authors contributed to the analysis of the data.

## Supporting information



Supporting Information

## Data Availability

The data that support the findings of this study are available from the corresponding author upon reasonable request.
