# Supplementary material for: A Universal and Versatile Zwitterionic Coating for Blood‐Contacting Catheters with Long Lengths and Complex Geometries
Source: Adv Sci (Weinh). 2025 Mar 24;12(19):2502411. doi: 10.1002/advs.202502411 (PMC12097014; doi:10.1002/advs.202502411)
Supplement: Supplementary file 1 — Supporting Information [file ADVS-12-2502411-s001.docx]

**Supporting Information**

**A Universal and Versatile Zwitterionic Coating for Blood-Contacting Catheters with Long Lengths and Complex Geometries**

Tong Zhang,^‡^ Tian Liang,^‡^ Qichao Pan, Shouyan Zhang, Shuhua Zhang, Zhi Geng*, Bo Zhu*

1. School of Materials Science and Engineering, Shanghai University, Shanghai 200444, China, 99 Shangda Road, Baoshan, Shanghai 200444

2. Shanghai Engineering Research Center of Organ Repair, Shanghai University, Shanghai 200444, China

3. Joint International Research Laboratory of Biomaterials and Biotechnology in Organ Repair, Ministry of Education

E-mail: bozhu@shu.edu.cn

**Keywords:** Catheter, Anticoagulant, Spontaneous coating, Phosphorylcholine, Antifouling


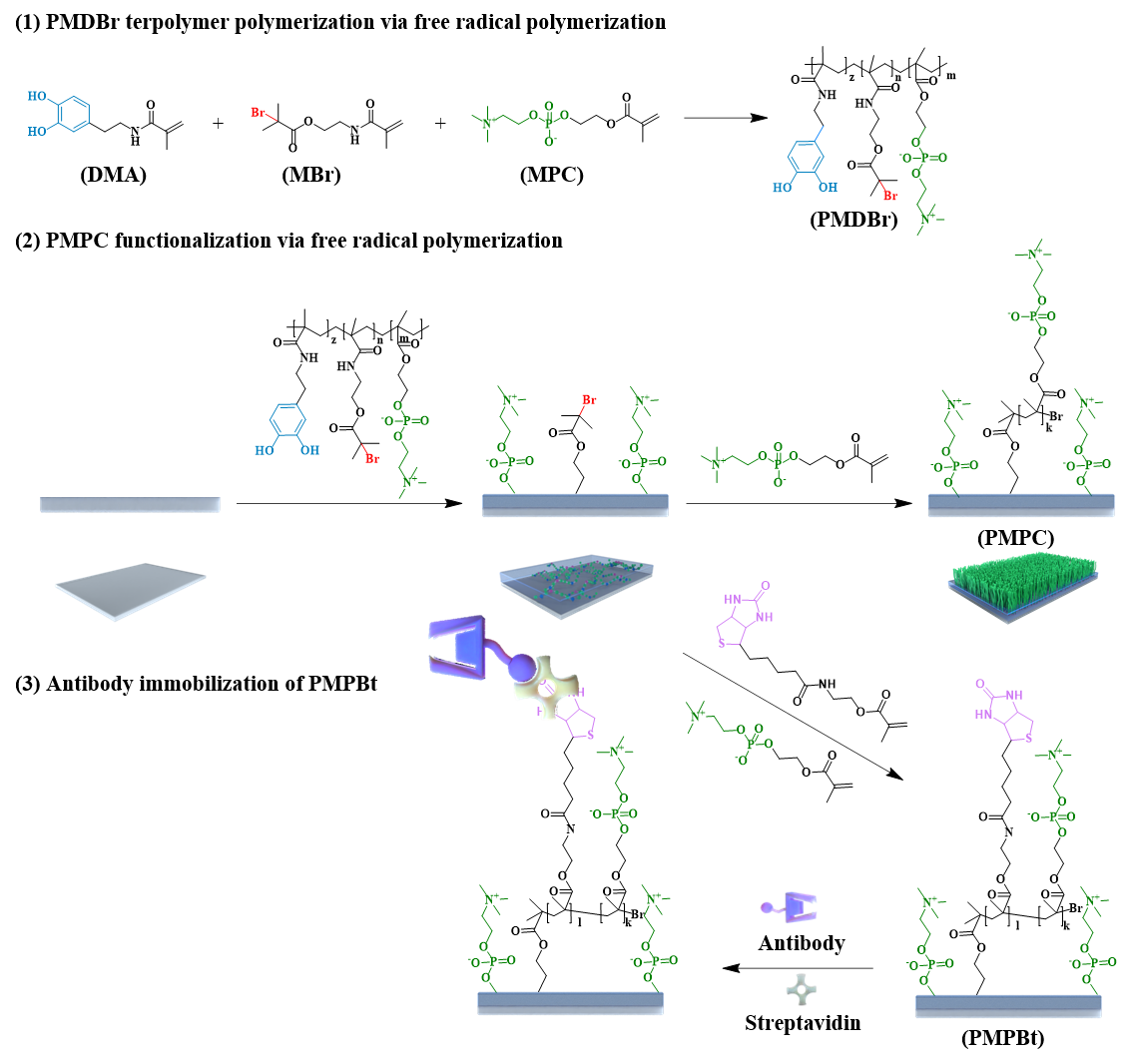


**Scheme S1**. Schematic illustration of the zwitterion coating s prepared from the PMDBr.


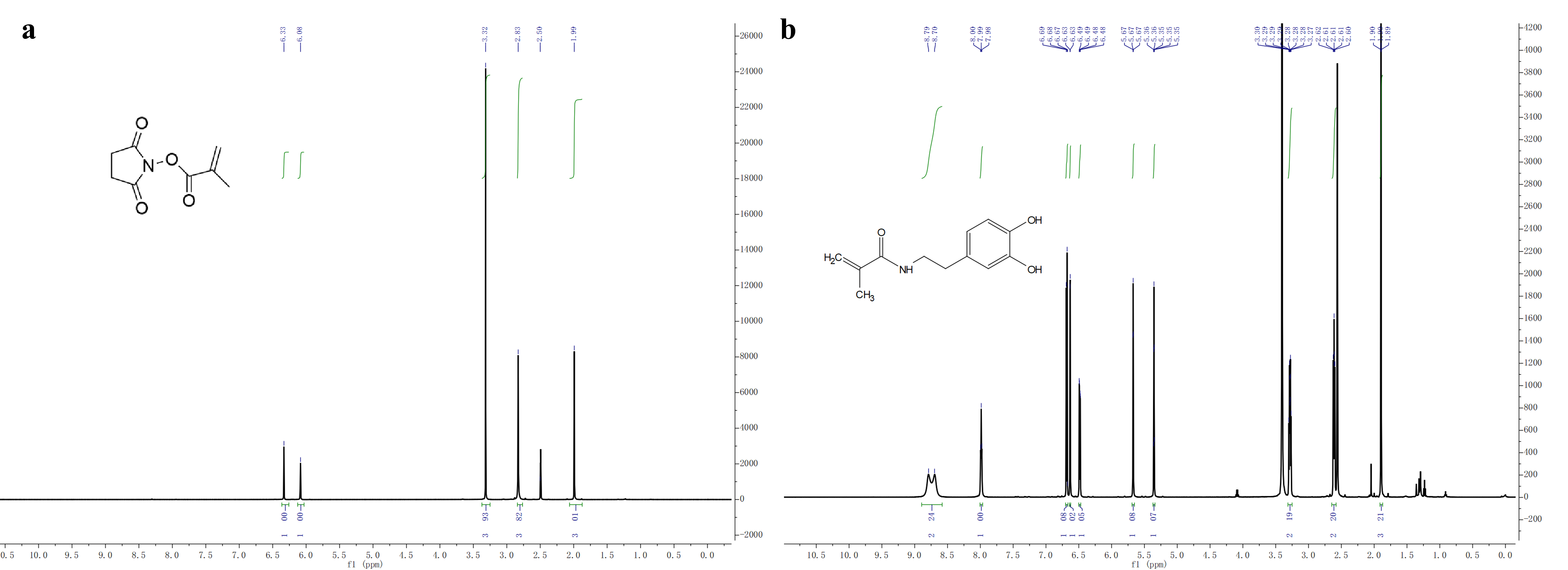

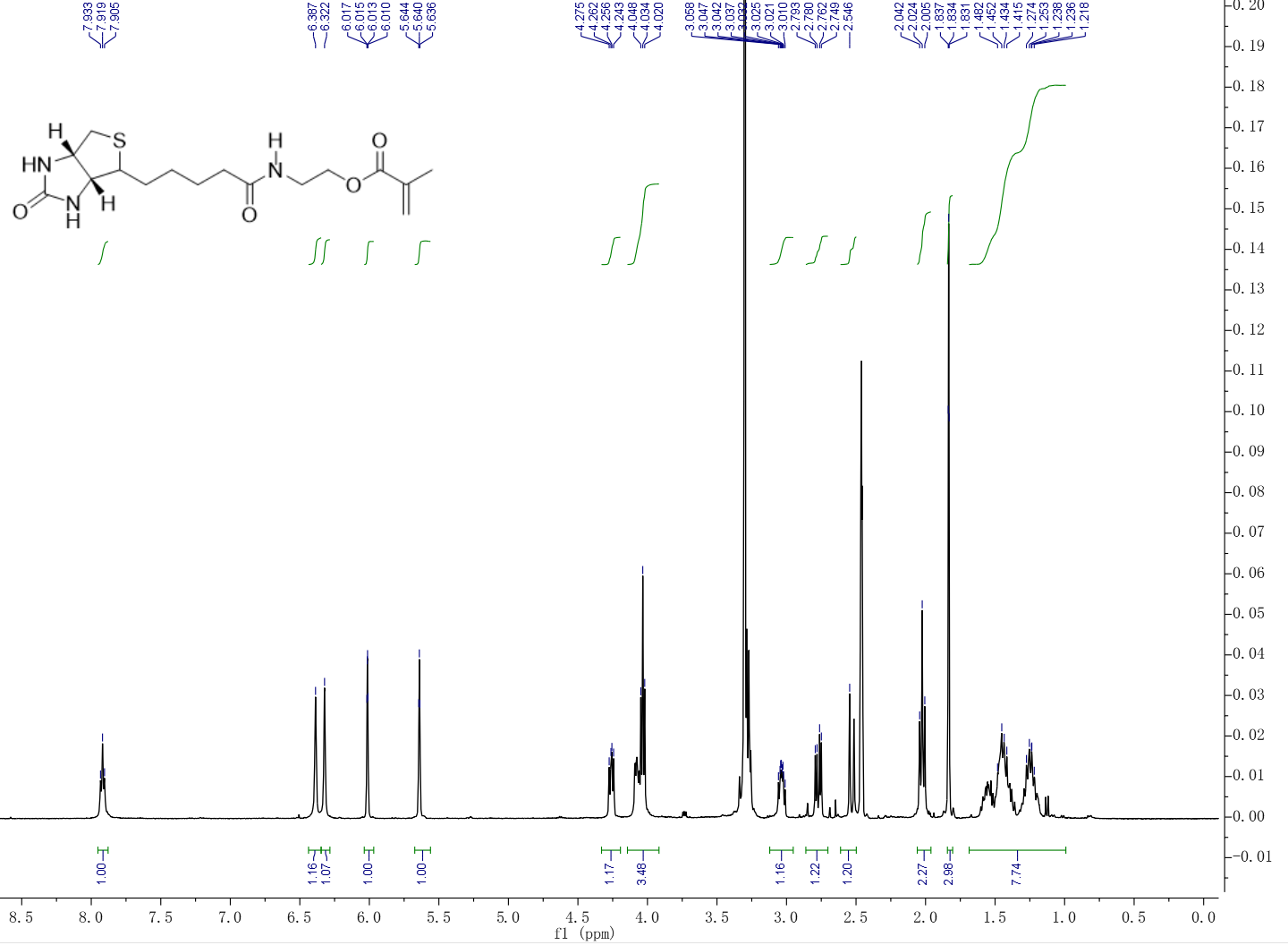


**Figure S1**. ^1^H NMR characteristic spectra of N-(3,4-Dihydroxyphenyl)ethyl methacrylamide (DMA) and biotin-functionalized methacrylate (MBt).

The PMDBr with multifunction was first prepared by free radical polymerization of N-(3,4-Dihydroxyphenyl) ethyl methacrylamide (DMA), 2-Methacryloxyethyl phosphorylcholine (MPC), and 2-(2-Bromoisobutyryl) Ethoxyl Methacrylamide (MBr) in DMF/H_2_O solution using AIBN as an initiator. By varying the molar ratio of MBr monomer (0%, 5%, and 10%, respectively), three PMDBr polymers (containing 0%, 5%, and 10%, respectively) were synthesized to demonstrate the controllability of the synthesis method proposed in this paper and to explore further how different monomer compositions affect the coating performance.

The ^1^H NMR and XPS results of the synthesized polymers are presented in **Figure S2** and **Figure 1c**. The characteristic signals of all units could be observed from the ^1^H NMR, and all peaks were well assigned to their chemical structures. The composition of the tricopolymers was confirmed by comparing the integrals of the benzyl protons in DMA (δ 6.78~6.43 ppm), N^+^-CH_2_- protons in MPC (δ 3.6~3.4 ppm), and Br-CH_2_-CH_3_ methyl protons in MBr (δ 1.8~1.7 ppm). The results are listed in Table S1. The successful synthesis of the polymers was confirmed again by XPS survey spectroscopy, and the MPC monomer content in the polymers was analyzed by deconvolution of the peaks in the N 1s level region, with results very close to that calculated from ^1^H NMR. The FT-IR spectra in Figure 1d illustrate the difference among polymers by different monomer compositions. The decrease in the proportion of three peaks at 1226 (P=O stretching), 1072 (P-O stretching), and 957 (N^+^-C stretching) in the full spectrum indicates that the phosphocholine groups are decreasing.^1^ The above results demonstrate the controllable synthesis of tricopolymers. The PSD of PDMBr in the pre-assembled solution exhibits a monodisperse pattern (**Figure S2b**). The particle size reaches a minimum of 5.65 nm when the MBr addition amount is 10%.


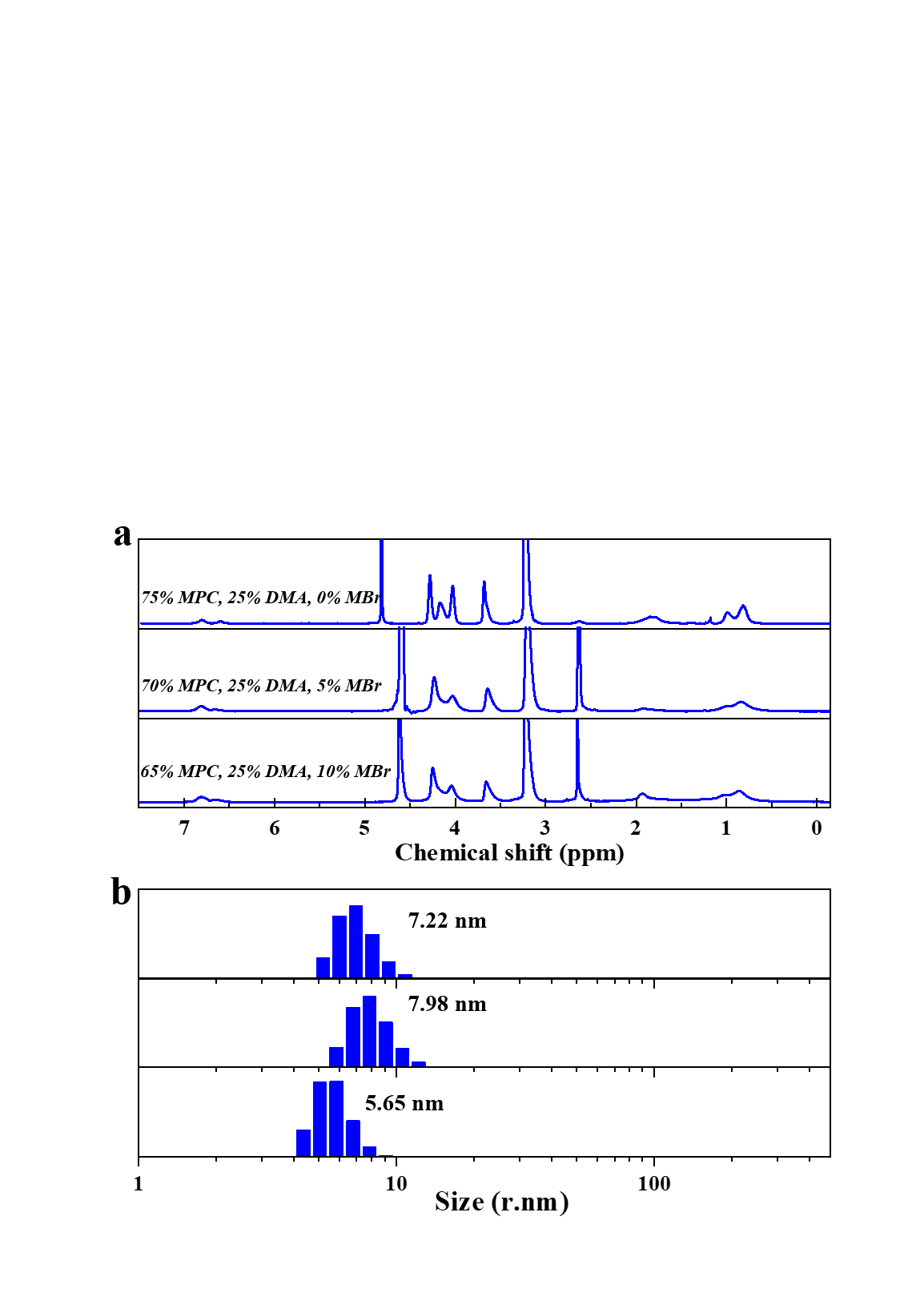


**Figure S2.** a) ^1^H NMR characteristic spectra and b) particle size distribution for the PMDBr polymers synthesized at varied MBr compositions(0%~10%) in their solutions in the mixture of DMSO and water (1:1) at a concentration of 1.5 mg/mL.

**Table S1.** P(DA-co-PC-co-Br) design and characteristics of the prepared copolymers

| **PMDBr** | **DMA: MPC: MBr**  **(in feed)** | **DMA: MBr: MPC**  **(^1^H NMR)^a^** | **PSD^b^**  **(nm)** |
| --- | --- | --- | --- |
| **0%MBr** | 25: 75: 0 | 26: 74: 0 | 7.33 |
| **5%MBr** | 25: 70: 5 | 22: 74: 4 | 7.98 |
| **10%MBr** | 25: 65: 10 | 25: 66: 9 | 5.65 |

**a Calculated from 1H NMR spectrum. b Measured by DLS.**

**Table S2.** The detailed data of the adhesion properties for PDBr, PMBr and the PMDBr polymer synthesized at varied MBr compositions(0%~10%).

| **Polymer** | **Monomer content**  **(DMA: MPC: MBr)** | **Deposition rate**  **Average(Hz/s)** | **Frequency change**  **Max(Hz)** |
| --- | --- | --- | --- |
| **PDBr** | 25%:**0%**:75% | 0.0056 | 8.12 |
| **PMBr** | **0%**:90%:10% | 0.02 | 14.22 |
| **PMDBr (0%MBr)** | 25%:75%:**0%** | 0.03 | 17.09 |
| **PMDBr (5%MBr)** | 25%:70%:5% | 0.12 | 31.38 |
| **PMDBr (10%MBr)** | 25%:65%:10% | 0.25 | 65.17 |


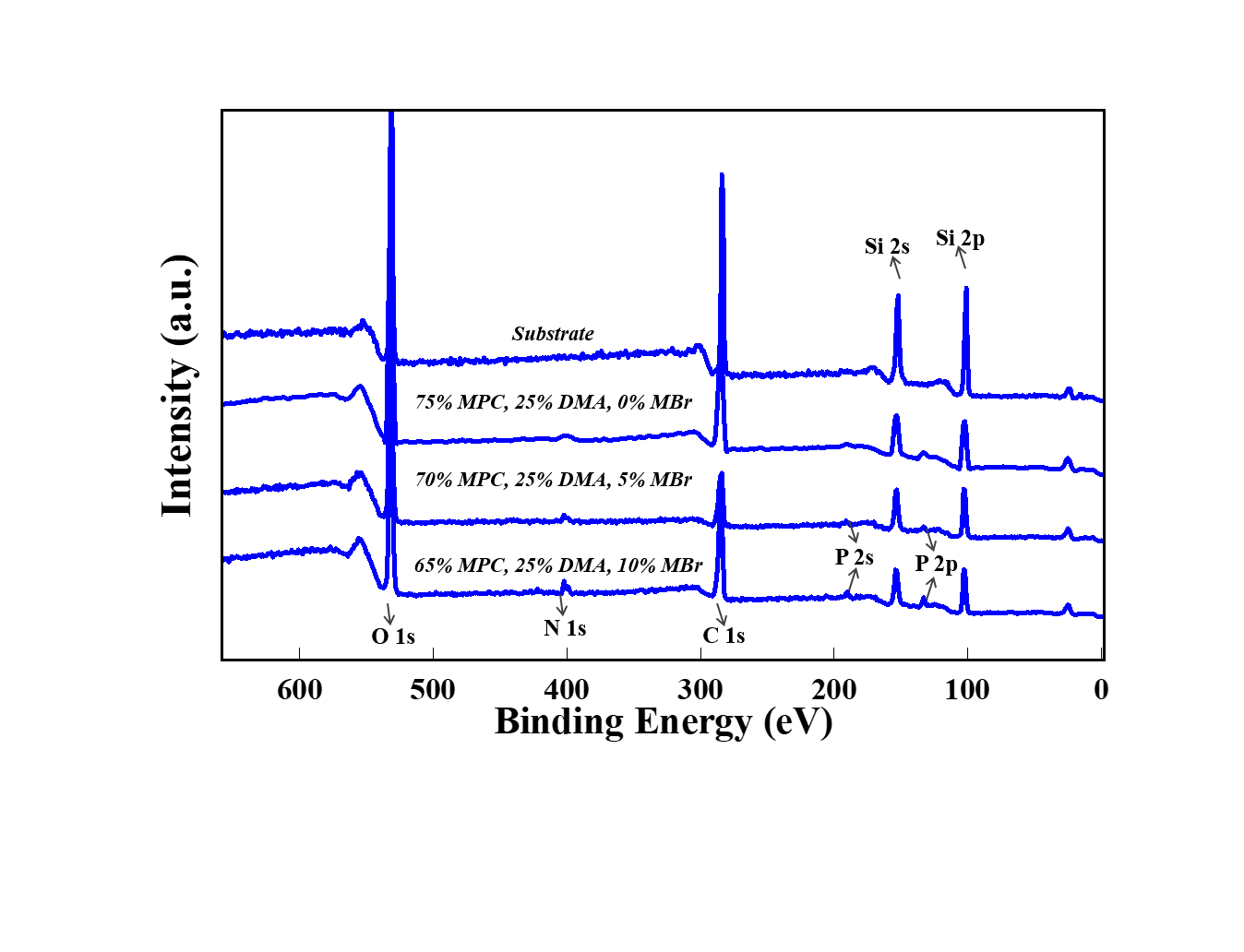


**Figure S3.** XPS survey spectra of the original Si substrates and those coated with the PMDBr polymer synthesized at varied MBr compositions(0%~10%).

**Table S3.** Element composition of the copolymers and those coated surfaces measured by XPS analysis

|  | **XPS composition (at%)** | | | |
| --- | --- | --- | --- | --- |
|  | **C 1s** | **N 1s** | **O 1s** | **P 2p** |
| **polymer-0%MBr** | 64.78 | 5.16 | 26.10 | 3.96 |
| **polymer-5%MBr** | 63.51 | 5.74 | 27.32 | 3.44 |
| **polymer-10%MBr** | 64.86 | 4.88 | 26.23 | 4.03 |
| **surface-0%MBr** | 36.72 | 5.28 | 56.50 | 1.49 |
| **surface -5%MBr** | 45.10 | 3.19 | 50.07 | 1.63 |
| **surface -10%MBr** | 57.95 | 4.19 | 34.65 | 3.21 |
| **surface grafted PMPC** | 59.28 | 6.17 | 30.03 | 4.53 |


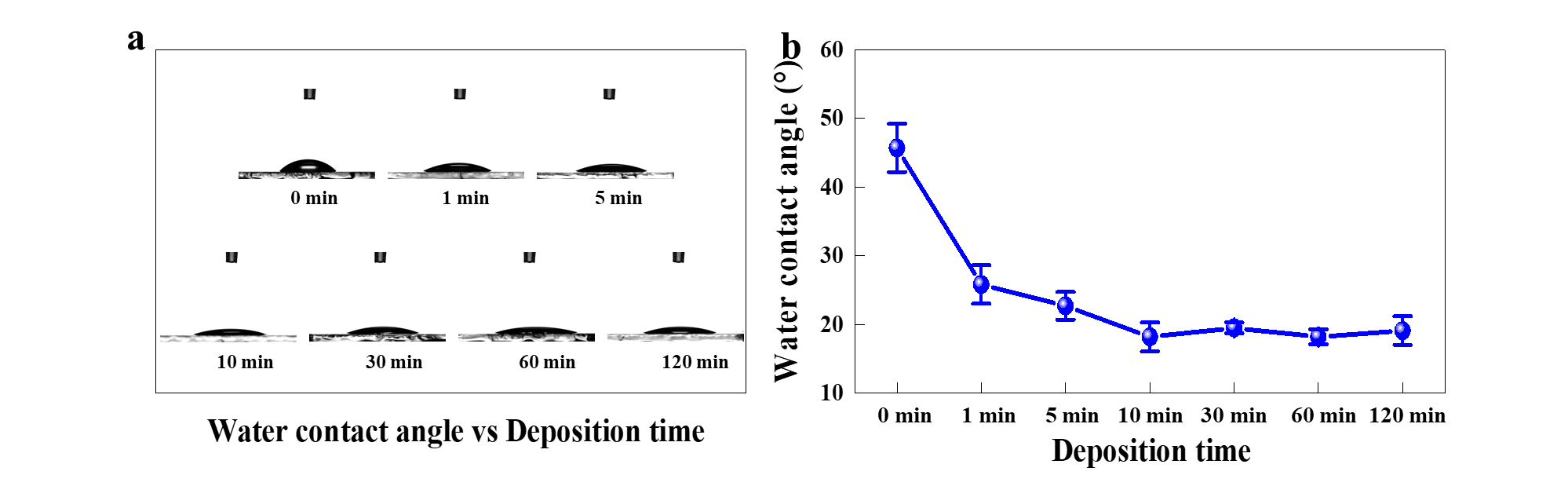


**Figure S4.** The Optical images of the water contact angle change of PMDBr polymers coating over the coating time.


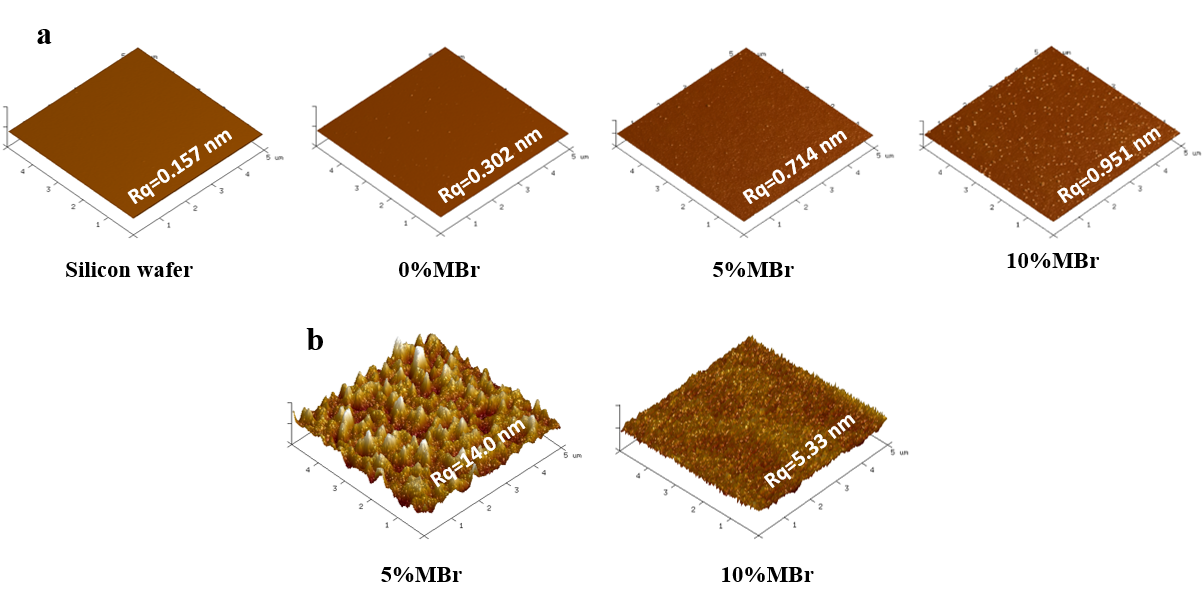


**Figure S5.** The AFM images and roughness parameters of (a) original Si substrates, the substrates coated with the PMDBr polymers synthesized at 0%, 5%, and 10% MBr compositions and (b) those further grafted by PMPC chains. The R_q_ represented the root mean square roughness. (n = 3).


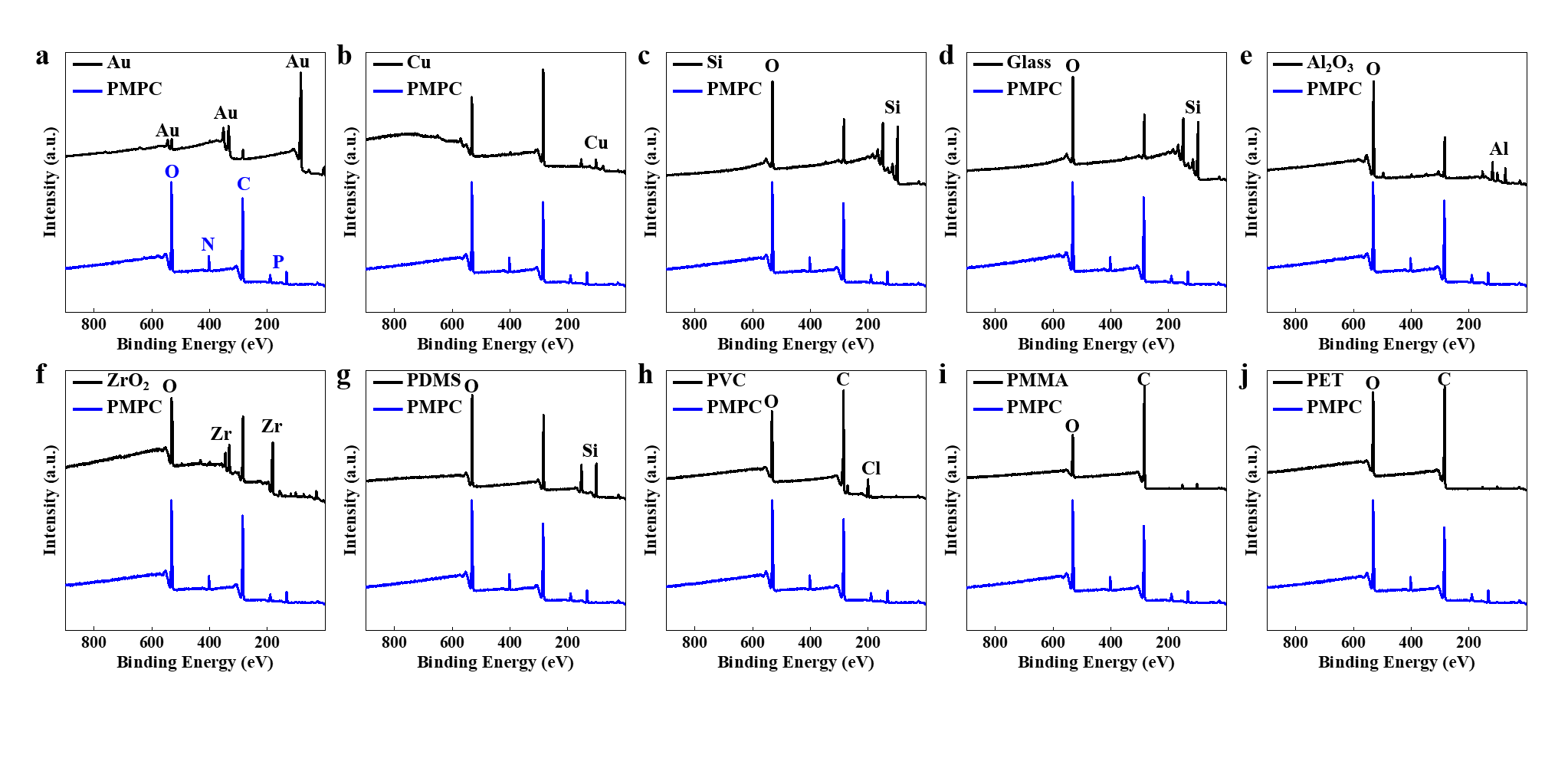


**Figure S6.** The XPS survey spectra of the PMPC coating on Au(a), Cu(b), Si(c), Glass(d), Al₂O₃(e), ZrO₂(f), PDMS(g), PVC(h), PMMA(i), and PET(j).


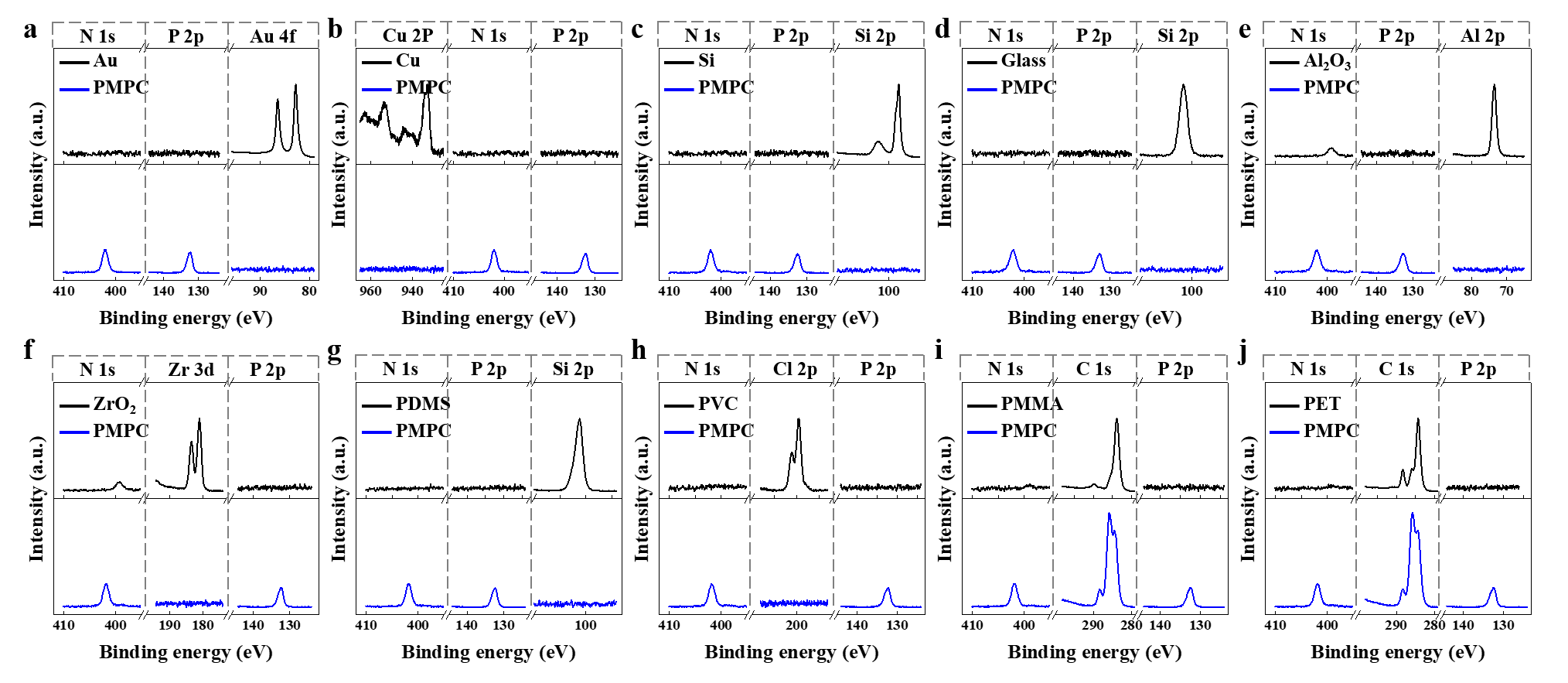


**Figure S7.** The High-resolution XPS spectra of the PMPC coating on Au(a), Cu(b), Si(c), Glass(d), Al₂O₃(e), ZrO₂(f), PDMS(g), PVC(h), PMMA(i), and PET(j).

**Table S4.** Element composition of the PMPC coatings on ten kinds of surfaces measured by XPS analysis

|  | **XPS composition (at%)** | | | |
| --- | --- | --- | --- | --- |
|  | **C 1s** | **N 1s** | **O 1s** | **P 2p** |
| **Bare Au** | 49.40 | 0 | 19.03 | 0 |
| **PMPC coating on Au** | 63.86 | 5.04 | 26.29 | 4.80 |
| **Bare Cu** | 76.98 | 0 | 18.76 | 0 |
| **PMPC coating on Cu** | 61.88 | 5.21 | 27.95 | 4.96 |
| **Bare Si** | 30.46 | 0 | 27.44 | 0 |
| **PMPC coating on Si** | 62.81 | 4.52 | 27.78 | 4.89 |
| **Bare Glass** | 42.55 | 0 | 40.06 | 0 |
| **PMPC coating on Glass** | 63.68 | 4.92 | 26.69 | 4.71 |
| **Bare Al_2_O_3_** | 35.67 | 2.47 | 41.35 | 0 |
| **PMPC coating on Al_2_O_3_** | 63.31 | 4.96 | 27.03 | 4.70 |
| **Bare ZrO_2_** | 34.12 | 2.58 | 34.12 | 0 |
| **PMPC coating on ZrO_2_** | 63.19 | 4.86 | 27.19 | 4.76 |
| **Bare PDMS** | 27.13 | 0 | 27.13 | 0 |
| **PMPC coating on PDMS** | 61.16 | 4.92 | 28.85 | 5.07 |
| **Bare PVC** | 75.32 | 0 | 24.68 | 0 |
| **PMPC coating on PVC** | 62.08 | 5.33 | 27.87 | 4.72 |
| **Bare PMMA** | 81.27 | 0 | 18.73 | 0 |
| **PMPC coating on PMMA** | 60.45 | 5.27 | 29.08 | 5.20 |
| **Bare PET** | 74.94 | 0 | 25.06 | 0 |
| **PMPC coating on PET** | 60.67 | 5.21 | 28.77 | 5.34 |


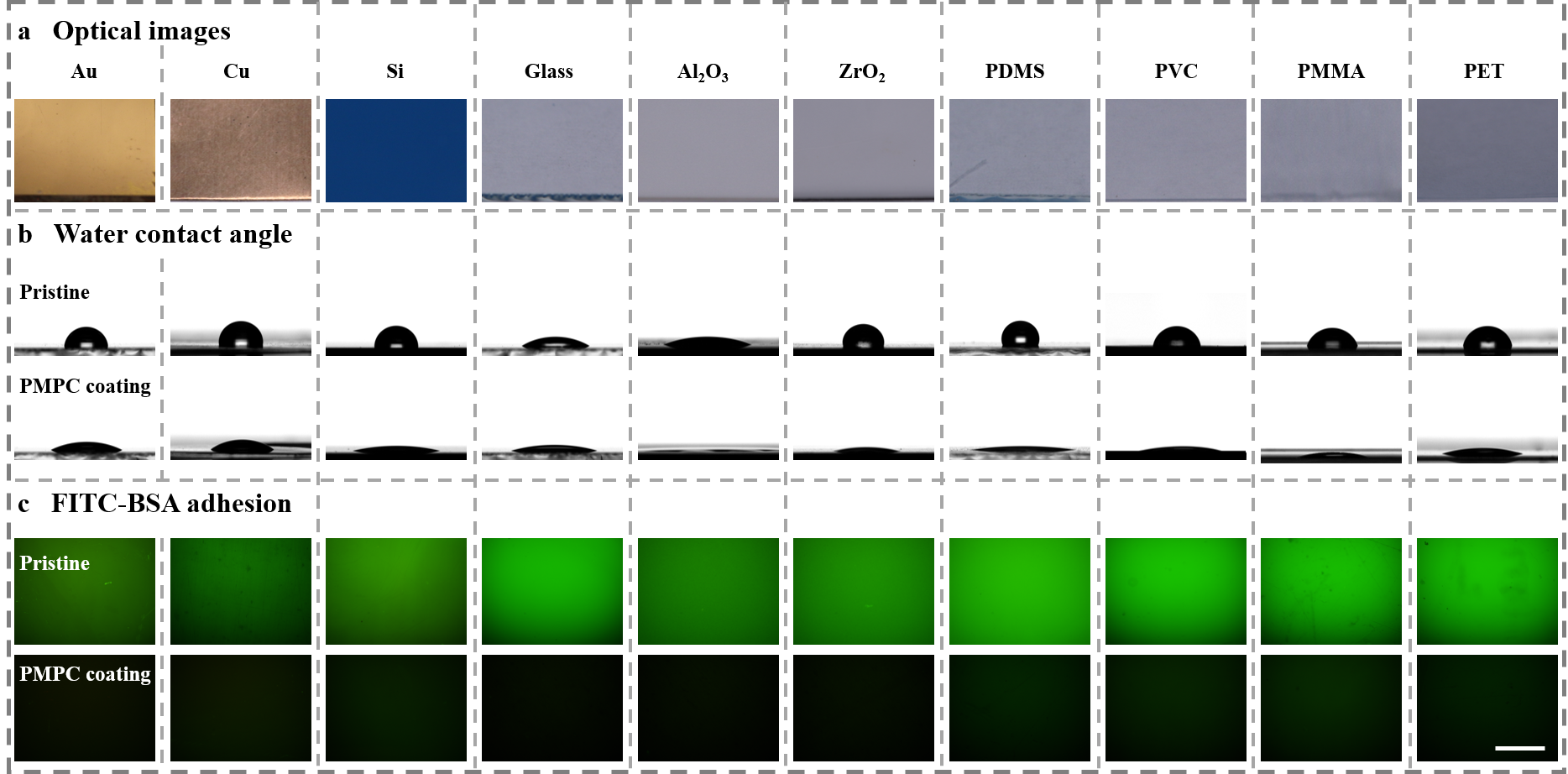


**Figure S8.** (a) The optical photograph of PMPC coatings on various substrates. (b) Static water contact angle in the air of multiple substrates.


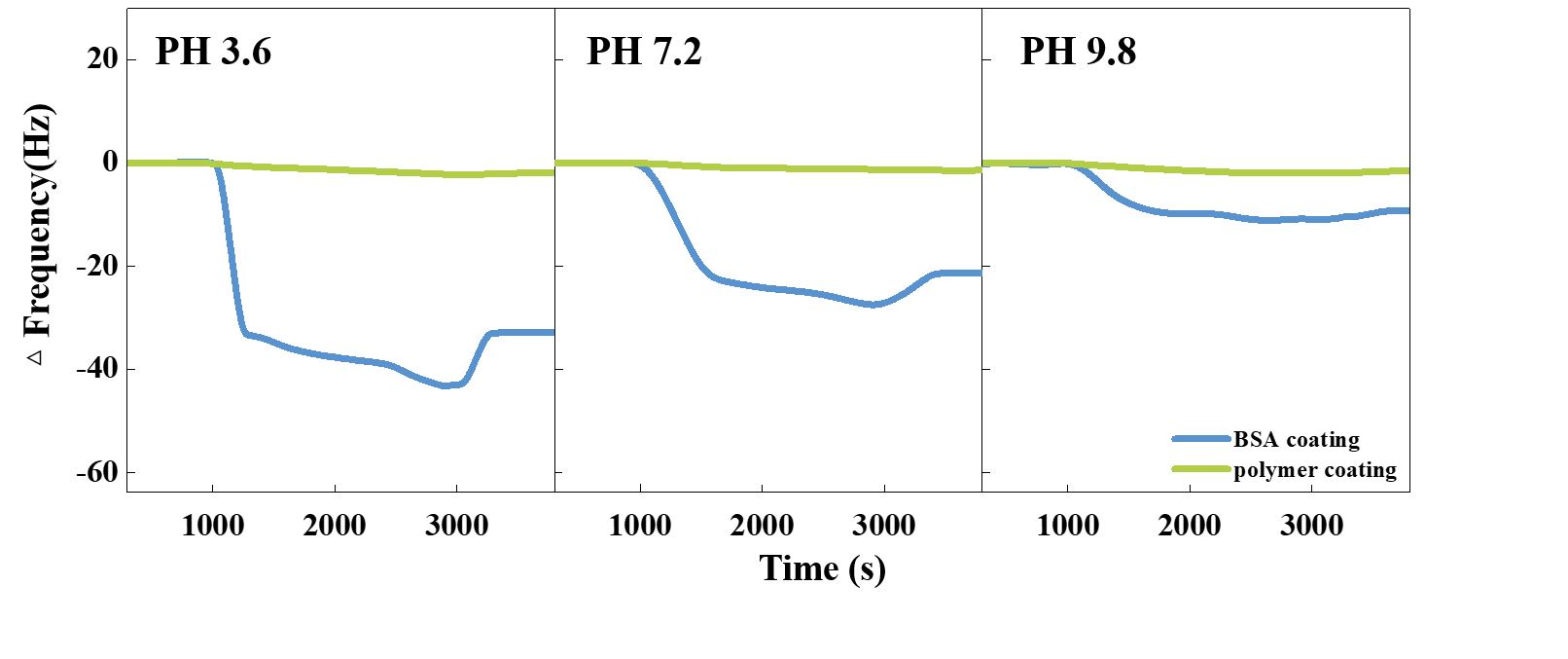


**Figure S9.** In situ monitoring of the frequency change induced by nonspecific interaction of the superhydrophillic zwitterionic (green) and the BSA (blue) coatings in the FBS solutions at various pH values.


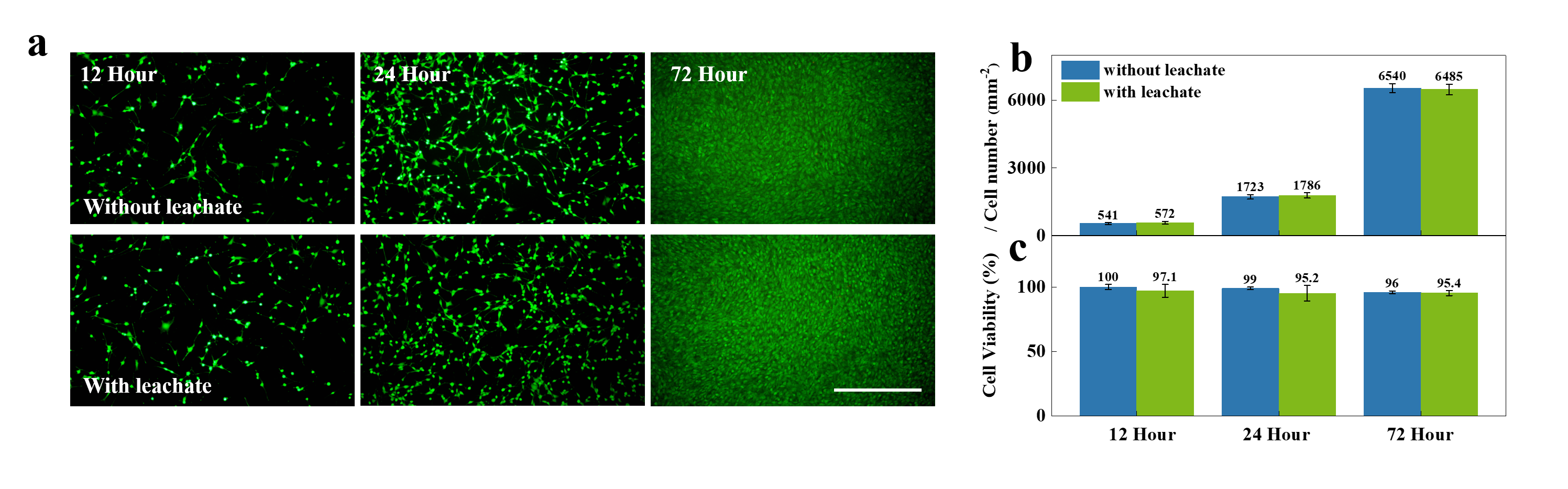


**Figure S10.** (a) Fluorescence microscopic images, after stained with live/dead kit, for NIH3T3 cells cultured for 12, 24, and 72 h in the medium with and without the leachate of PMPC grafted coating. The scale bar is 300 μm. (b) The cell density and (c) viability of NIH3T3 cells cultured for 12, 24, and 72 h with and without leachate of PMPC grafted coating. The bars represent the mean ± SD (n = 3).


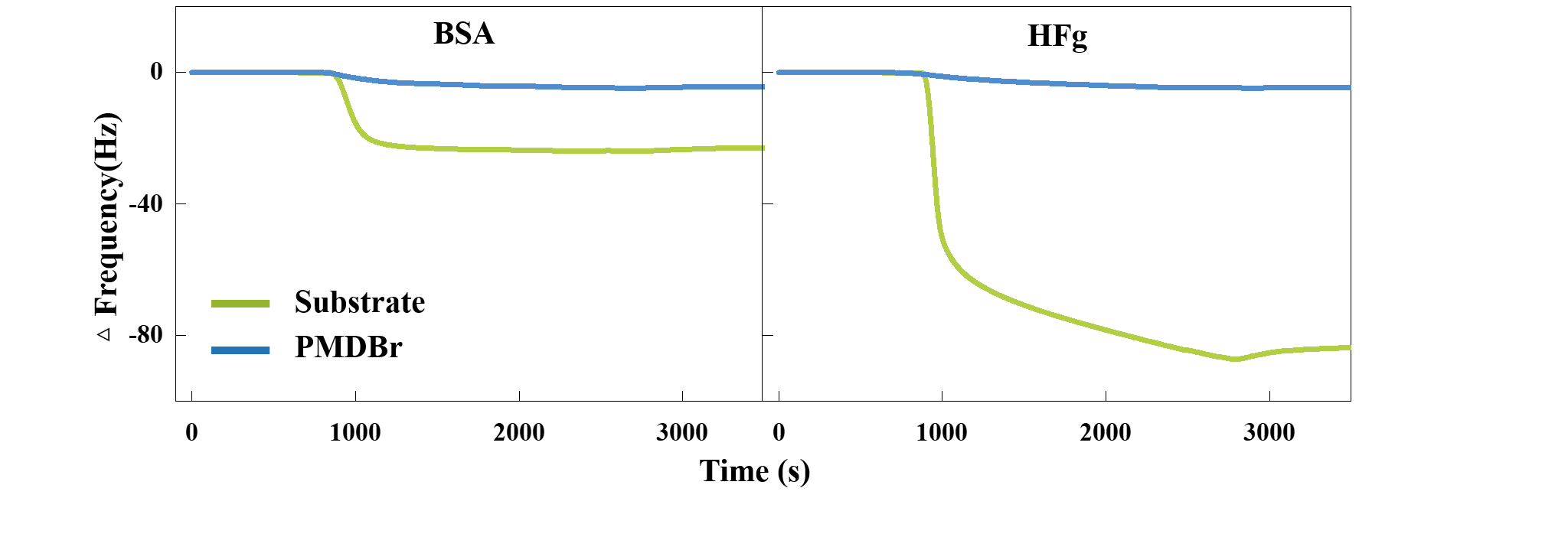


**Figure S11.** In situ monitoring of the Δf resulted from the nonspecific BSA (left) and HFg (right) adsorptions of PMDBr coating. The uncoated gold QCM crystal was included as a control.


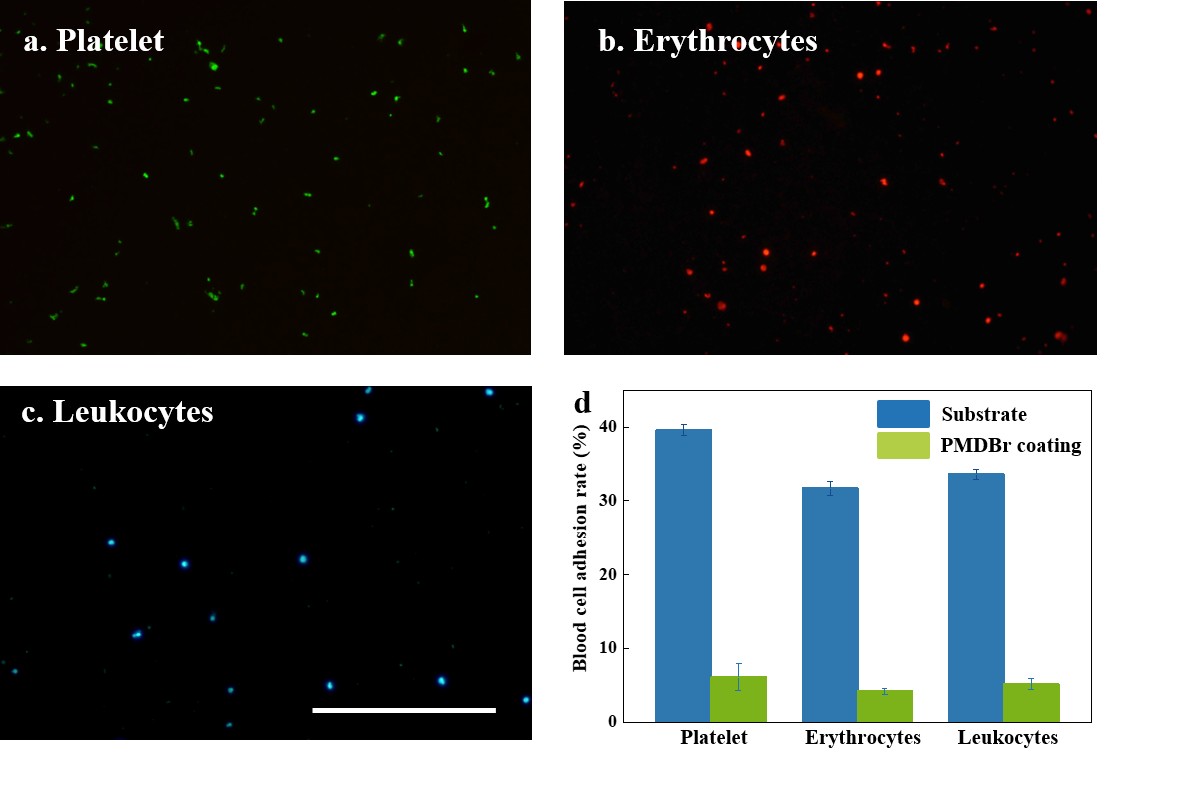


**Figure S12.** Fluorescence microscopic images, after seeded platelets (a), erythrocytes (b), and leukocytes (c) for 3 h, for the pristine glass coverslip and PMDBr coating. The scale bar is 300 μm. (d) Cell adhesion rates of platelet, erythrocytes, and leukocytes on the pristine glass coverslip and PMDBr coating. The bars represent the mean ± SD (n = 3).


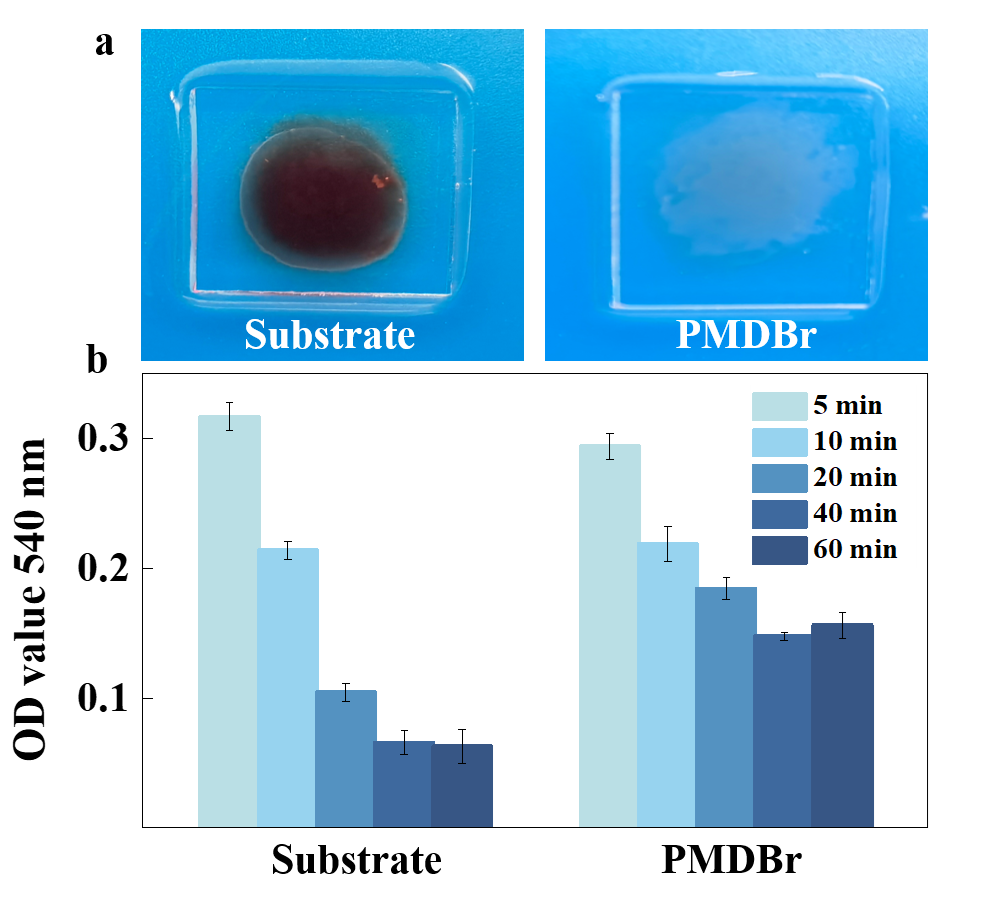


**Figure S13.** Optical images of the blood clots formed within 60 min blood incubation on the pristine glass coverslip and PMDBr coating.


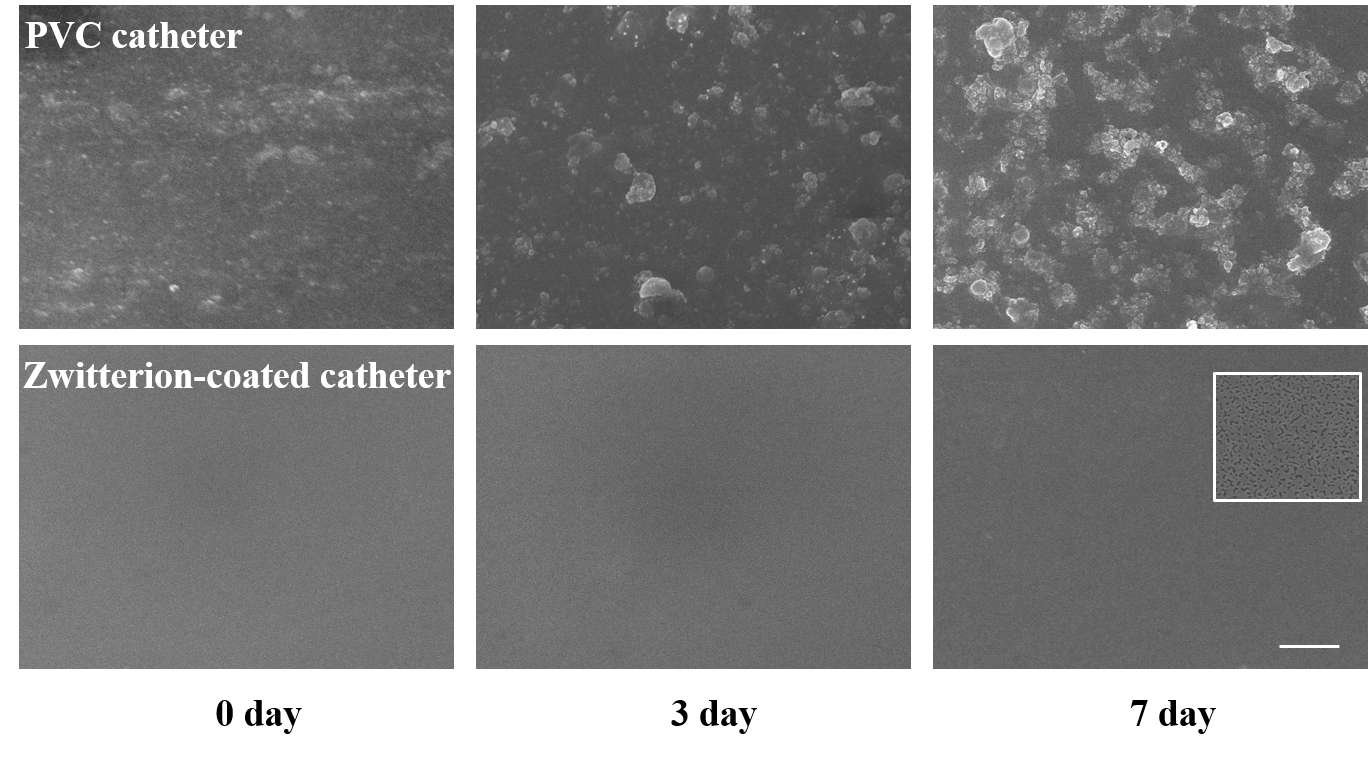


**Figure S14.** SEM images of PVC catheter and zwitterion coating catheter after circulation in SPSS for 0, 3, and 7 days. The scale bar is 3 μm.


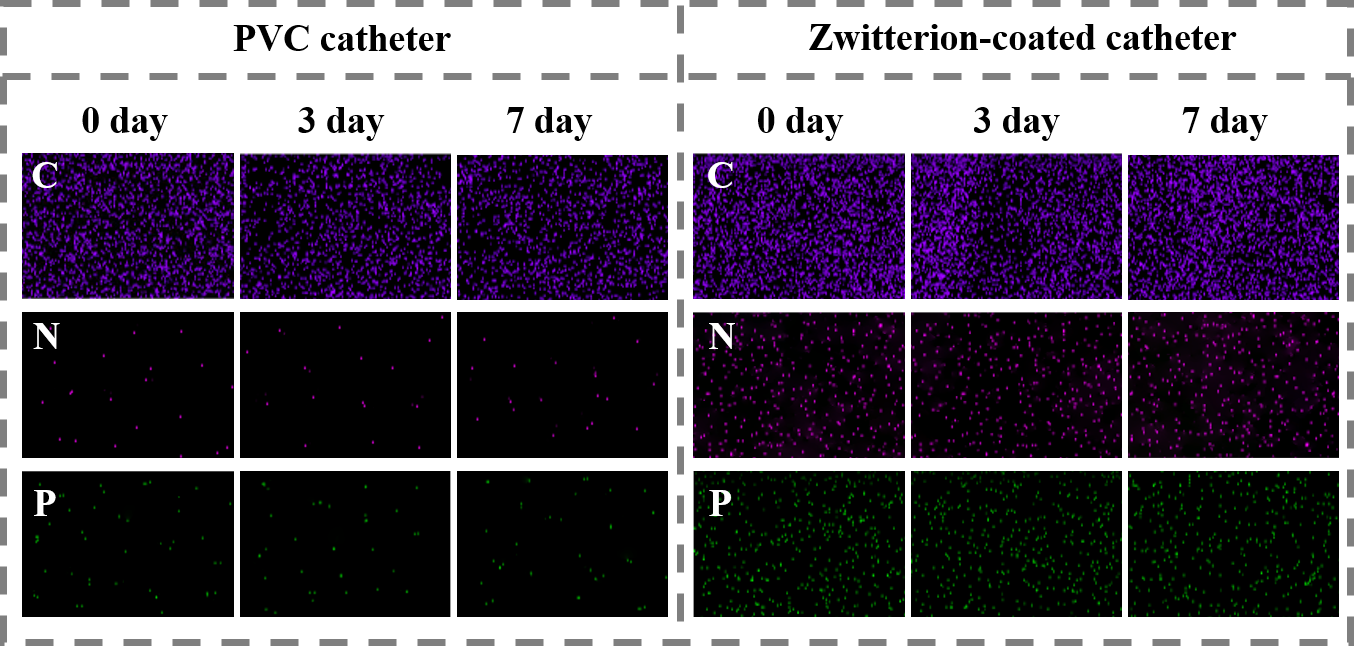


**Figure S15.** EDS mapping of PVC catheter and zwitterion coating catheter after circulation in SPSS for 0, 3, and 7 days.


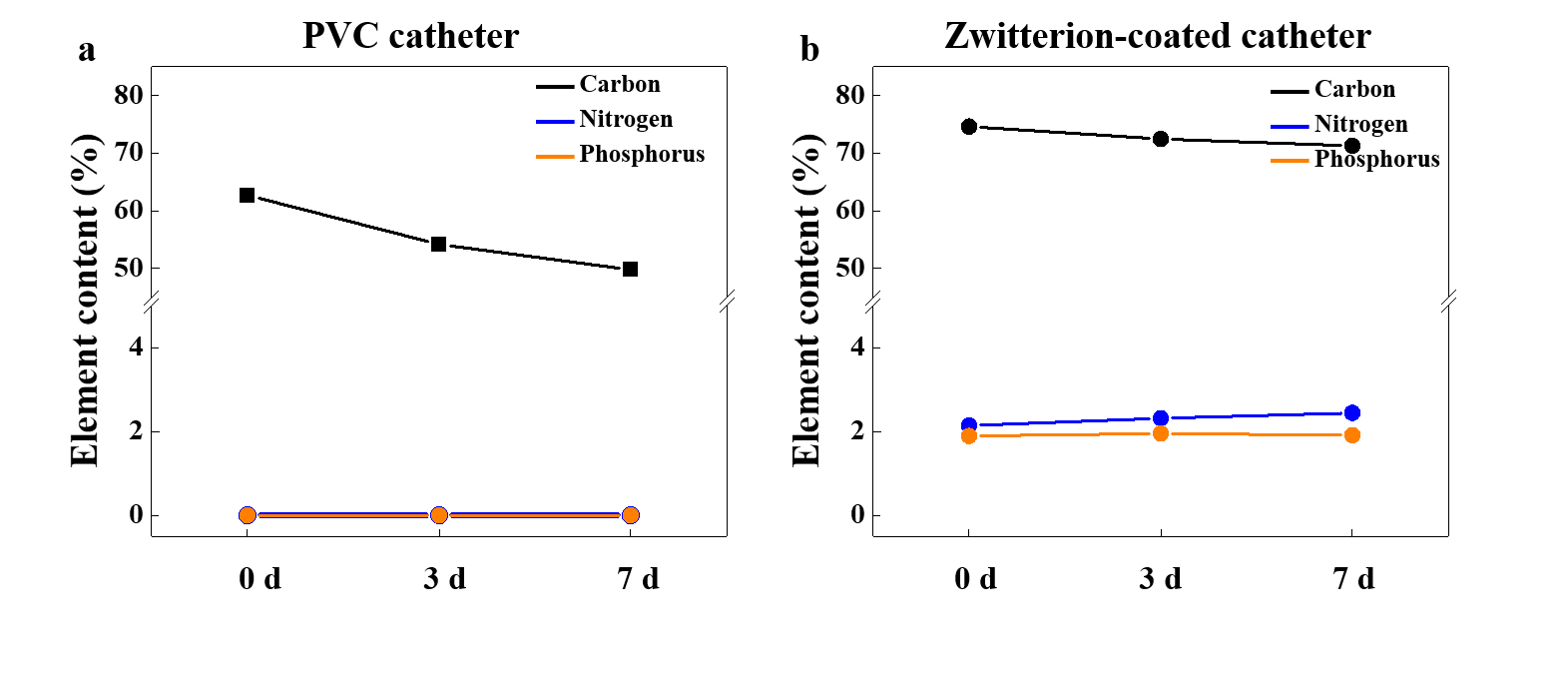


**Figure S16.** Changes of C, N and P element content of inner walls of both PVC catheter and zwitterion coating catheter after circulation in SPSS for 0, 3, and 7 days.


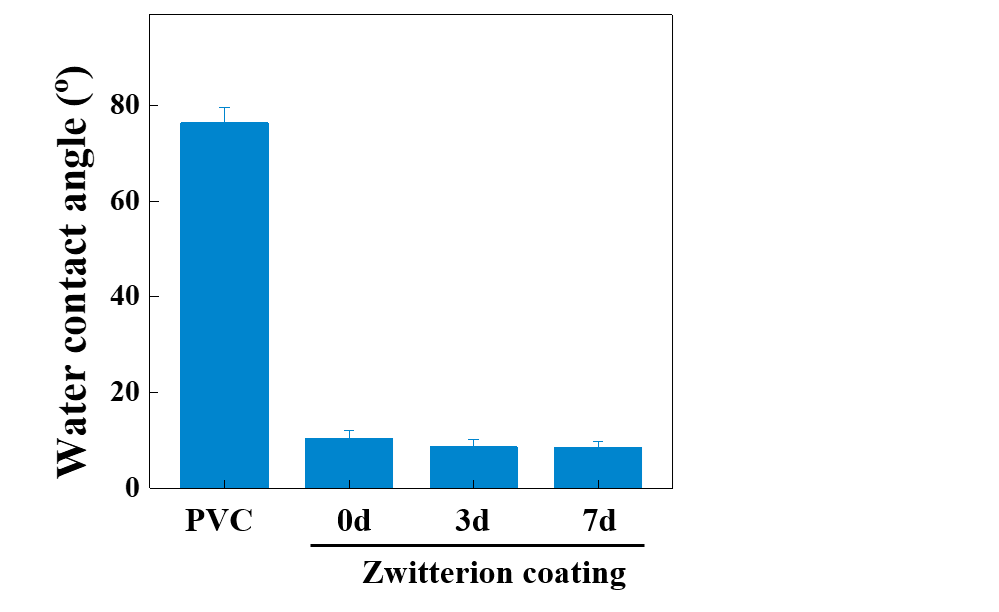


**Figure S17.** WCA of zwitterion coating catheter after circulation in SPSS for 0, 3, and 7 days.


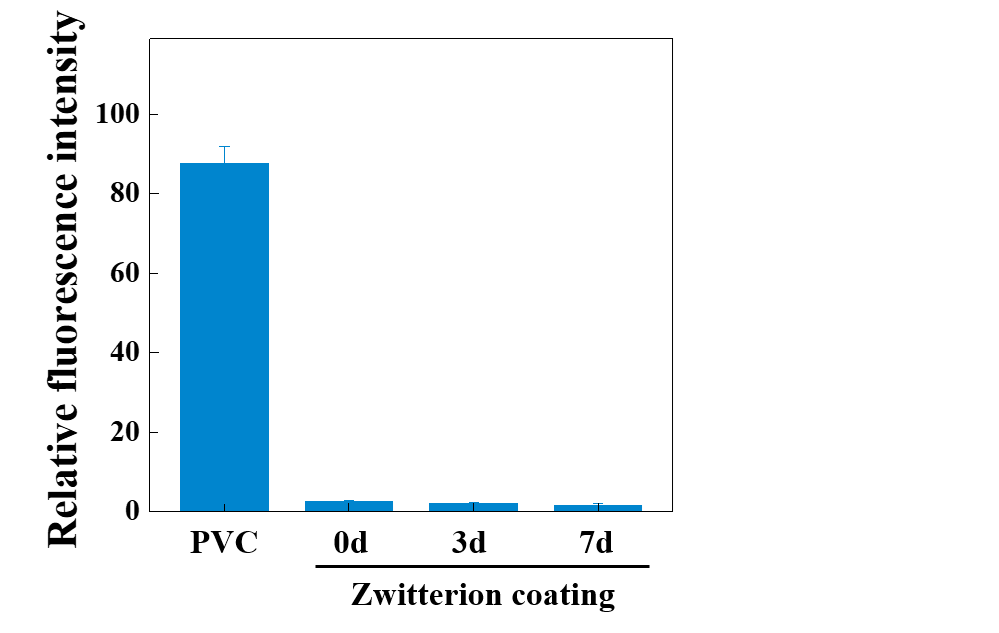


**Figure S18.** The normalized relative fluorescence intensity of zwitterion coated catheter after exposure to the FITC-BSA solution. Before tests, these catheters were subjected to the SPSS circulation for 0, 3, and 7 days.


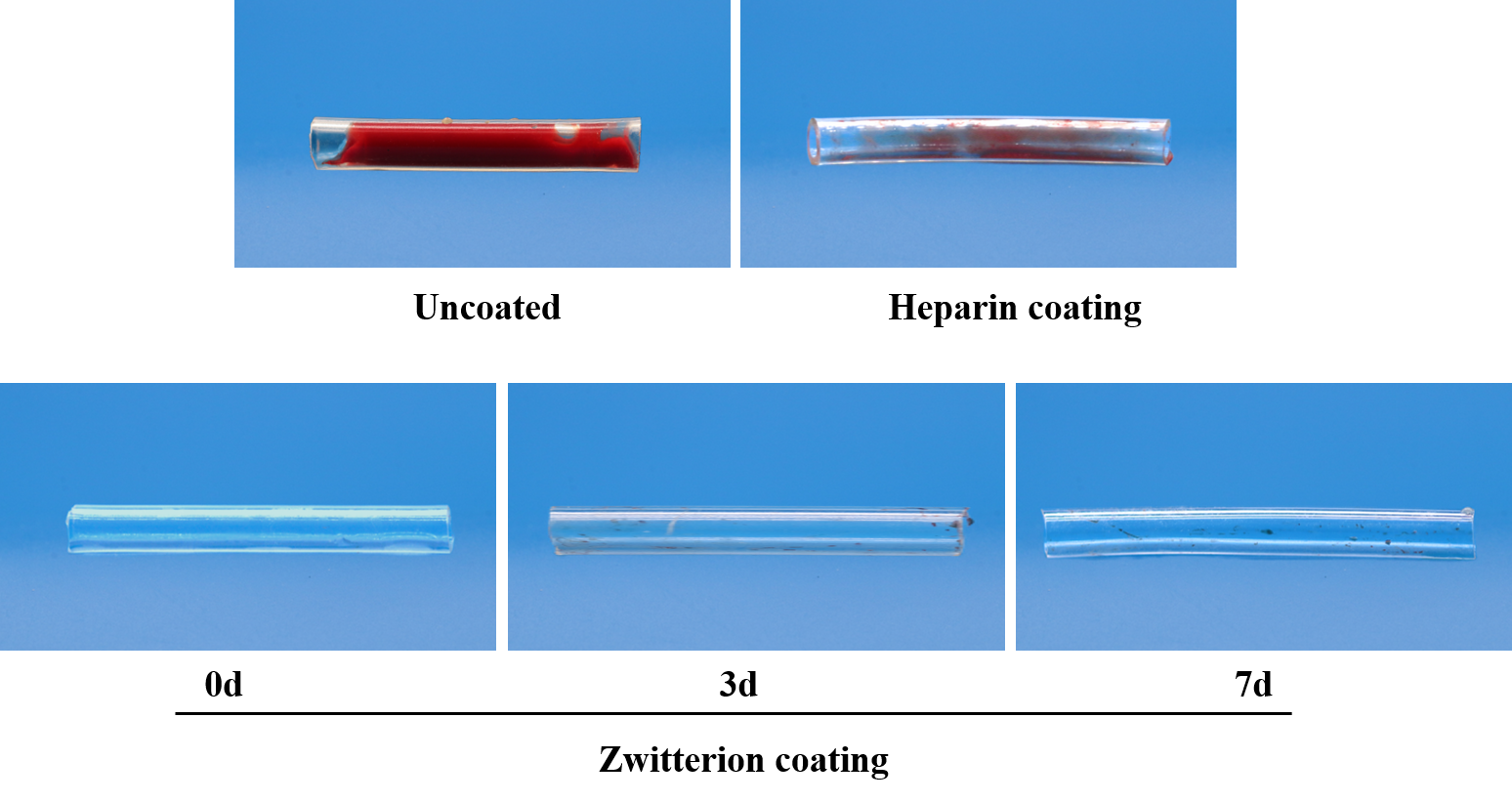


**Figure S19.** The optical images, after tested by the in vitro blood flow system, for the pristine, heparin-coated and zwitterion-coated catheter.


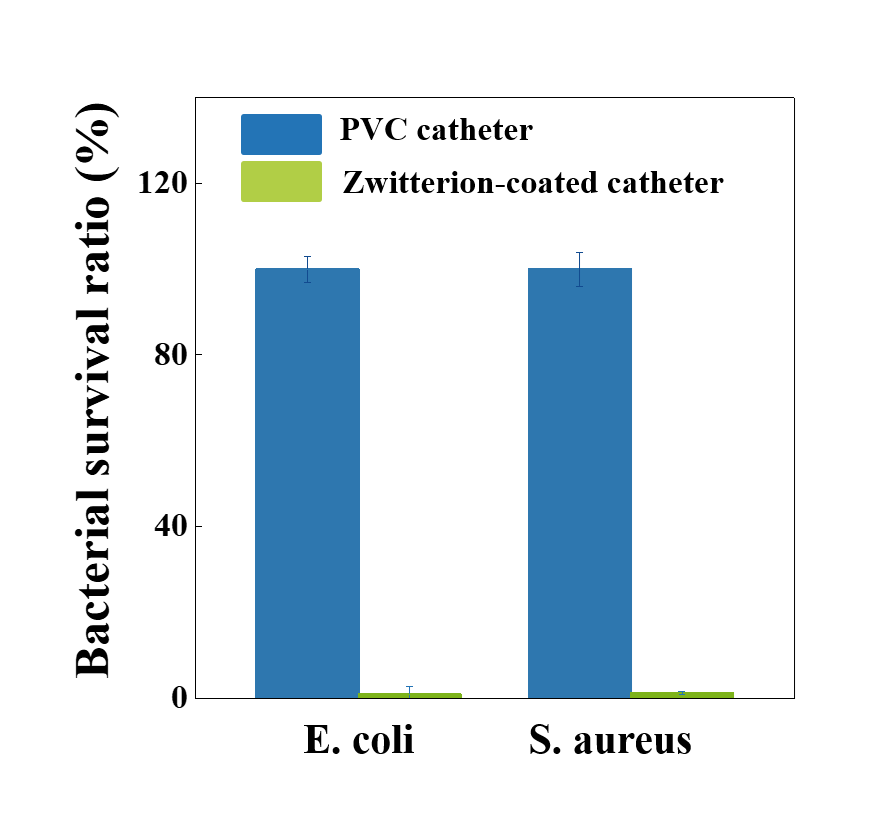


**Figure S20.** Bacterial survival ratio of the PVC catheter and zwitterion-coated catheter.


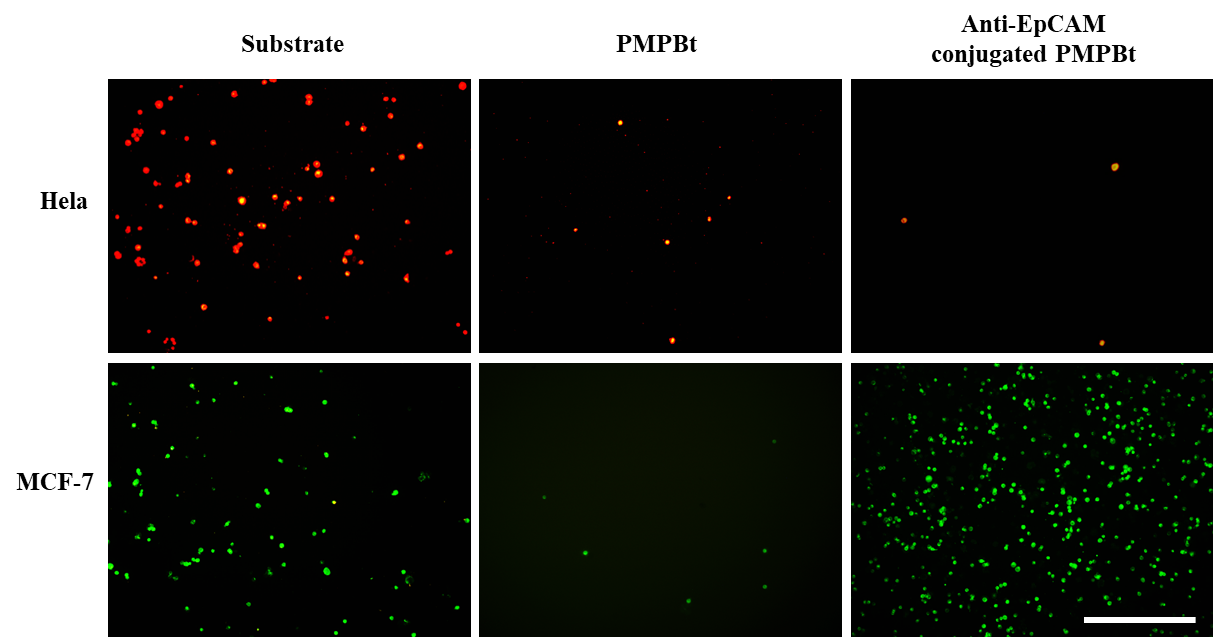


**Figure S21.** Fluorescence microscopic images, after seeded MCF-7 cells (green) and Hela cells (red) for 2 h, for the pristine, the copolymer of MPC and MBt grafted, and the anti-EpCAM conjugated the copolymer of MPC and MBt modified PVC substrates. The scale bar is 300 μm.


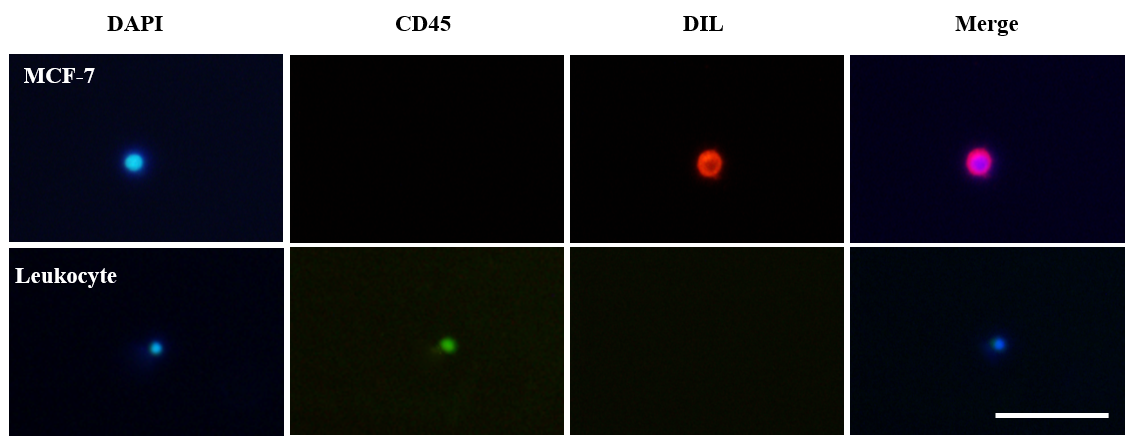


**Figure S22.** Fluorescence image of MCF-7 cells and leukocytes captured on the anti-EpCAM conjugated the copolymer of MPC and MBt modified PVC catheters. MCF-7 showed DAPI+/DIL+/anti-CD45- and WBCs were DAPI and anti-CD45 positive only. The scale bar is 50 μm.

**References**

1. Wiarachai, O.; Vilaivan, T.; Iwasaki, Y.; Hoven, V. P., Clickable and Antifouling Platform of Poly[(propargyl methacrylate)-ran-(2-methacryloyloxyethyl phosphorylcholine)] for Biosensing Applications. *Langmuir* **2016,** *32* (4), 1184-1194.
